# New Bifunctional Chelators Incorporating Dibromomaleimide Groups for Radiolabeling of Antibodies with Positron Emission Tomography Imaging Radioisotopes

**DOI:** 10.1021/acs.bioconjchem.0c00710

**Published:** 2021-03-16

**Authors:** Matthew Farleigh, Truc Thuy Pham, Zilin Yu, Jana Kim, Kavitha Sunassee, George Firth, Nafsika Forte, Vijay Chudasama, James R. Baker, Nicholas J. Long, Charlotte Rivas, Michelle T. Ma

**Affiliations:** † School of Biomedical Engineering and Imaging Sciences, King’s College London, St. Thomas’ Hospital, London SE1 7EH, U.K.; ‡ Department of Chemistry, University College London, 20 Gordon Street, London WC1H 0AJ, U.K.; § Department of Chemistry, Imperial College London, Molecular Sciences Research Hub, London W12 0BZ, U.K.

## Abstract

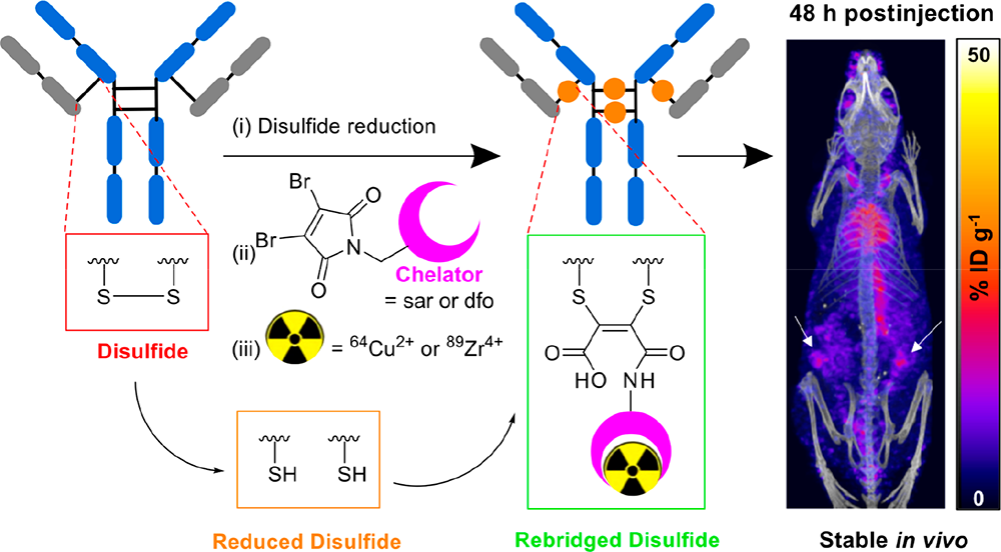

Positron Emission Tomography (PET) imaging with antibody-based contrast agents frequently uses the radioisotopes [^64^Cu]Cu^2+^ and [^89^Zr]Zr^4+^. The macrobicyclic chelator commonly known as sarcophagine (sar) is ideal for labeling receptor-targeted biomolecules with [^64^Cu]Cu^2+^. The siderophore chelator, desferrioxamine-B (dfo), has been widely used to incorporate [^89^Zr]Zr^4+^ into antibodies. Here, we describe new bifunctional chelators of sar and dfo: these chelators have been functionalized with dibromomaleimides (dbm), that enable site-specific and highly stable attachment of molecular cargoes to reduced, solvent-accessible, interstrand native disulfide groups. The new sar–dbm and dfo–dbm derivatives can be easily conjugated with the IgG antibody trastuzumab via reaction with reduced interstrand disulfide groups to give site-specifically modified dithiomaleamic acid (dtm) conjugates, sar–dtm–trastuzumab and dfo–dtm–trastuzumab, in which interstrand disulfides are rebridged covalently with a small molecule linker. Both sar– and dfo–dtm–trastuzumab conjugates have been radiolabeled with [^64^Cu]Cu^2+^ and [^89^Zr]Zr^4+^, respectively, in near quantitative radiochemical yield (>99%). Serum stability studies, *in vivo* PET imaging, and biodistribution analyses using these radiolabeled immunoconjugates demonstrate that both [^64^Cu]Cu-sar–dtm–trastuzumab and [^89^Zr]Zr-dfo–dtm–trastuzumab possess high stability in biological milieu. Dibromomaleimide technology can be easily applied to enable stable, site-specific attachment of radiolabeled chelators, such as sar and dfo, to native interstrand disulfide regions of antibodies, enabling tracking of antibodies with PET imaging.

Monoclonal IgG antibody therapies have been transformative in the treatment of many cancers and autoimmune diseases, and new antibody therapies are likely to have further clinical impact. The ability to quantitatively image antibody biodistribution at the whole-body level can help predict individual patient response, as well as aid in understanding treatment outcomes during clinical development.^1,2^ Positron-emitting radiometallic isotopes attached to antibodies via chelators have been widely used to image antibody biodistribution using Positron Emission Tomography (PET).

Conventional labeling strategies have attached chelators to antibodies using electrophilic groups such as isothiocyanates, *N*-hydroxysuccinimides, and anhydrides that react with solvent accessible primary amines of lysine side chains.^3−5^ These bioconjugation methods are simple, but as these reactions are not site-specific and lack stoichiometric control, the presence of multiple lysine side chains leads to heterogeneous product mixtures. The resulting chelator–antibody conjugates often demonstrate reduced affinity for target receptors, particularly at high chelator:antibody conjugation ratios.^6−8^ Several recent and elegant site-specific strategies include enzyme-mediated conjugation of chelators to (i) glycan regions of IgG antibodies using a combination of β-1,4-galactosidase/β-1,4-galactosyltransferase(Y289L),^9−11^ (ii) hinge region glutamine residues of IgG antibodies using a combination of deglycosylation with *N*-glycosidase followed by chelator coupling with bacterial transglutaminase,^12^ and (iii) modified C-termini of antibody fragment derivatives using the bacterial enzyme sortase A.^13^ Alternatively, incorporation of a non-natural amino acid containing an azide group in an IgG antibody has enabled orthogonal and site-selective conjugation of chelators using click chemistry.^14^ These approaches have resulted in radiolabeled chelator–protein conjugates that display improved PET imaging capabilities relative to radiolabeled conjugates prepared using conventional, non-site-specific methods. However, these enzyme-based or protein engineering-based conjugation methods are more difficult to implement than conventional, non-site-specific conjugation technologies. Furthermore, modification or removal of glycan regions can modify the pharmacokinetics of antibodies, as well as affect their Fc-receptor binding properties and antibody-dependent cell-mediated toxicity.^15−17^

Maleimide derivatives have been widely used to incorporate radiolabeled chelators into IgG antibodies and other proteins.^18−21^ Maleimides react selectively with reduced disulfides at near-neutral pH, and in the case of IgG antibodies, reduction of the four solvent-accessible, interstrand disulfide bonds leads to the presence of eight available thiol attachment points. While maleimides enable site-selective attachment of chelators and other cargoes to IgG antibodies, these conjugates are unstable: the resulting thioether can undergo a retro-Michael reaction in biological milieu, converting back to the starting thiol and maleimide.^21−24^*In vivo*, the starting maleimide motif, still tethered to its payload, can then react with endogenous molecules containing bioavailable thiols, such as glutathione and albumin. In radionuclide imaging, this can potentially result in accumulation of radioactivity at off-target sites, decreasing image contrast, sensitivity, and the ability to quantify antibody distribution.

New conjugation platforms including bissulfone,^25^ divinylpyrimidine,^26^ divinyltriazine,^27^ dibromoalkyl oxetane,^28^ dibromopyridazinedione,^29,30^ and disubstituted maleimide^31−36^ derivatives enable site-specific attachment of cargo to dithiol groups of antibodies, including those at IgG hinge regions. Here, we focus on dibromomaleimide: this motif reacts specifically with two reduced thiol groups of antibodies, thus enabling concomitant attachment of cargo *and* rebridging of two cysteines. The resulting dithiomaleimide can be hydrolyzed to a dithiomaleamic acid under mildly basic conditions to give homogeneous antibody conjugates.^32−35^ Importantly, the dithiomaleamic acid conjugates are unreactive toward serum thiols and do not undergo retro-Michael reactions in biological media, unlike conventional maleimide derivatives. This dibromomaleimide platform has the potential to enable highly stable site-specific radiolabeling of antibodies at hinge region disulfides.

Zirconium-89 (*t*_1/2_ = 78 h, β^+^*E*_max_ = 897 keV, 23%) and copper-64 (*t*_1/2_ = 12.7 h, β^+^*E*_max_ = 656 keV, 18%) have both been used for imaging antibody distribution with PET: the half-lives of these isotopes match the time required for antibodies to clear circulation and accumulate in target tissue (1 day–1 week). Derivatives of a naturally occurring siderophore, desferrioxamine-B (dfo), are commonly used to incorporate [^89^Zr]Zr^4+^ into monoclonal IgG antibodies for clinical and preclinical PET imaging.^1,2,4,11,18,19^ The dfo chelator contains three hydroxamate groups, enabling hexadentate *O*_6_ coordination of Zr^4+^. Many chelators have been developed for [^64^Cu]Cu^2+^ over the past three decades. Peptide and protein conjugates of the hexaazamacrobicyclic chelator, 3,6,10,13,16,19-hexaazabicyclo[6.6.6]icosane, known commonly as “sarcophagine” (sar), have proved to be simple to radiolabel and are highly stable to demetalation *in vivo*, leading to high quality PET images.^3,10,13,37−39^ Here we describe preparation of dibromomaleimide derivatives of dfo and sar chelators and show the usability and applicability of this dibromomaleimide platform for site-specific radiolabeling of an IgG1 antibody with [^89^Zr]Zr^4+^ and [^64^Cu]Cu^2+^.

## Preparation of sar–dbm and dfo–dbm Bifunctional Chelators

**Scheme 1:**
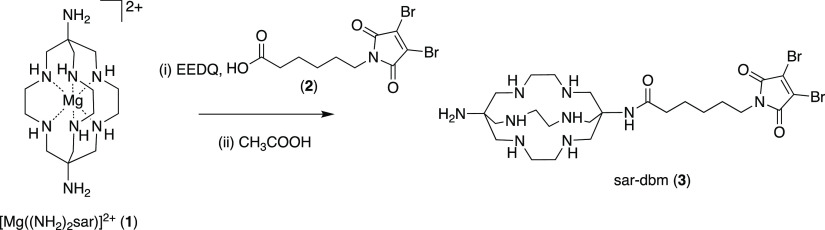
Synthesis of sar–dbm

**Scheme 2:**
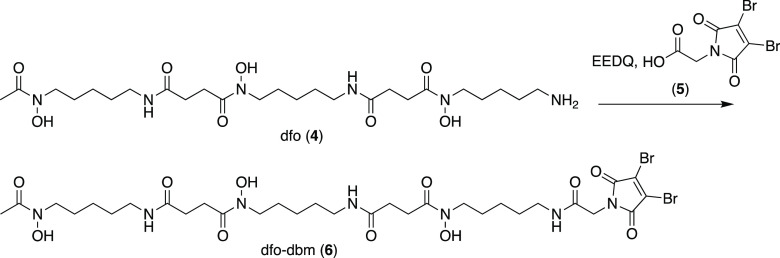
Synthesis of dfo–dbm

The 1,8-diamino-3,6,10,13,16,19-hexaazabicyclo[6.6.6]icosane, or “(NH_2_)_2_sar”, chelator is a useful precursor for preparation of bioconjugates. It contains two primary amines and six secondary amines, all of which are reactive toward electrophiles.^40^ Prior studies have achieved selective functionalization of primary amines by “protecting” secondary amine groups through metal ion (Cu^2+^ or Mg^2+^) complexation.^37−39^ We elected to use [Mg((NH_2_)_2_sar)]^2+^ (**1**) as a precursor,^39^ as following derivatization, Mg^2+^ can be easily removed from the chelator by acidification. [Mg((NH_2_)_2_sar)]^2+^ was reacted with 3,4-dibromomaleimide-*N*-hexanoic acid (**2**) and coupling agent *N*-ethoxycarbonyl-2-ethoxy-1,2-dihydroquinoline (EEDQ) in acetonitrile (Scheme 1). The resulting product mixture was treated with acetic acid, prior to purification using reverse-phase HPLC, to yield **sar–dbm** (**3**) in 8% yield. Similarly, dfo (**4**) was reacted with dibromomaleimide-*N*-glycine (**5**) and EEDQ in dimethyl sulfoxide, followed by purification using reverse-phase HPLC to give **dfo–dbm** (**6**, Scheme 2) in 22% yield.

The inclusion of a C_6_ aliphatic linker group in sar–dbm aided reverse-phase HPLC purification and isolation of sar–dbm: the sar chelator motif is very hydrophilic and is poorly retained on reverse-phase columns in the absence of hydrophobic appendages. In contrast, the dfo chelator had sufficient adsorption on reverse-phase stationary phases, and so a C_2_ linker group, which has demonstrated improved conjugation properties relative to a C_6_ linker (*vide infra*),^34^ was utilized.

The yield of sar–dbm (8%) was significantly lower than the yield of dfo–dbm (22%). We attribute this to the lower reactivity of the apical primary amine groups of (NH_2_)_2_sar and its complexes relative to other primary amines, possibly due to steric encumbrance of the macrobicyclic hexaamine rings. Previously reported syntheses, in which (NH_2_)_2_sar complexes have been reacted with electrophilic motifs, have required significant heating.^37−40^ Here, the reaction was not heated, as the dibromomaleimide group is sensitive to heat. Although the yield of sar–dbm was low under these reaction conditions, it could be obtained in a one-pot synthesis from [Mg((NH_2_)_2_sar)]^2+^.

The compound **sar–dbm**_**2**_, in which both apical primary amines of (NH_2_)_2_ sar react with 2 equiv of 3,4-dibromomaleimide-*N*-hexanoic acid to yield a sarcophagine chelator containing two dibromomaleimide groups, was also observed. Although we have detected and even isolated small amounts of sar–dbm_2_ and its Mg^2+^ complex, [Mg(sar–dbm_2_)]^2+^, analyses of crude reaction mixtures revealed that it was present in low yield (see the Supporting Information).

## Radiolabeled sar–dtm–Trastuzumab and dfo–dtm–Trastuzumab

The monoclonal antibody trastuzumab was chosen as a model antibody for assessing conjugation of sar–dbm and dfo–dbm: trastuzumab targets the human epidermal growth factor receptor 2 (HER2) and is widely used for treatment of HER2-positive breast cancer. The four solvent-accessible interstrand disulfide bonds of trastuzumab were reduced with tris(2-carboxyethyl)phosphine) (TCEP), the reduced antibody was reacted with 8 mol equiv of either sar–dbm or dfo–dbm at pH 8.5, and the conjugation reactions were monitored over time by SDS-PAGE (*vide infra*). These reaction conditions were adapted from well-established methods for coupling drug-dibromomaleimide or fluorophore-dibromomaleimides to IgG1 constructs.^31,34,41^ Notably, increasing the molar equivalents of chelator–dbm relative to the trastuzumab antibody, for example, from 8 mol equiv of sar–dbm to either 16 or 24 mol equiv of sar–dbm, did not significantly affect the efficacy of the conjugation reaction (Figure S1).

**Scheme 3:**
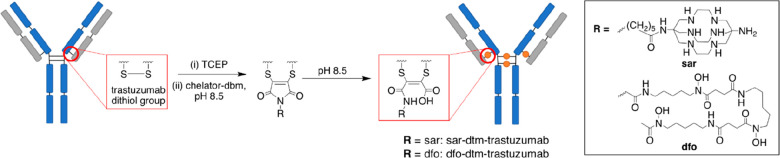
Preparation of Chelator–dtm–Trastuzumab Immunoconjugates

For reaction of either sar–dbm or dfo–dbm with reduced trastuzumab, thiol substitution to give dithiomaleimide conjugates proceeded rapidly (within 5 min). Subsequent hydrolysis of the dithiomaleimide to the dithiomaleamic acid (dtm) was achieved by further incubation of the reaction solutions at pH 8.5 for 2–48 h (Scheme 3). Prior studies on this class of compounds have shown that dithiomaleimide hydrolysis proceeds faster in the presence of electron-withdrawing imide substituents: significantly, the proximity of the amide bond to the maleimide motif increases the rate of hydrolysis.^34^ For dfo–dtm–trastuzumab, with a C_2_ glycine-derived linker, hydrolysis (as monitored by SDS-PAGE) was complete within 2 h (Figure S2). The sar–dtm–sarcophagine conjugate, with a longer C_6_ aminohexanoate linker, was incubated for 48 h at 37 °C to ensure complete hydrolysis (Figure S1).

**Figure 1:**
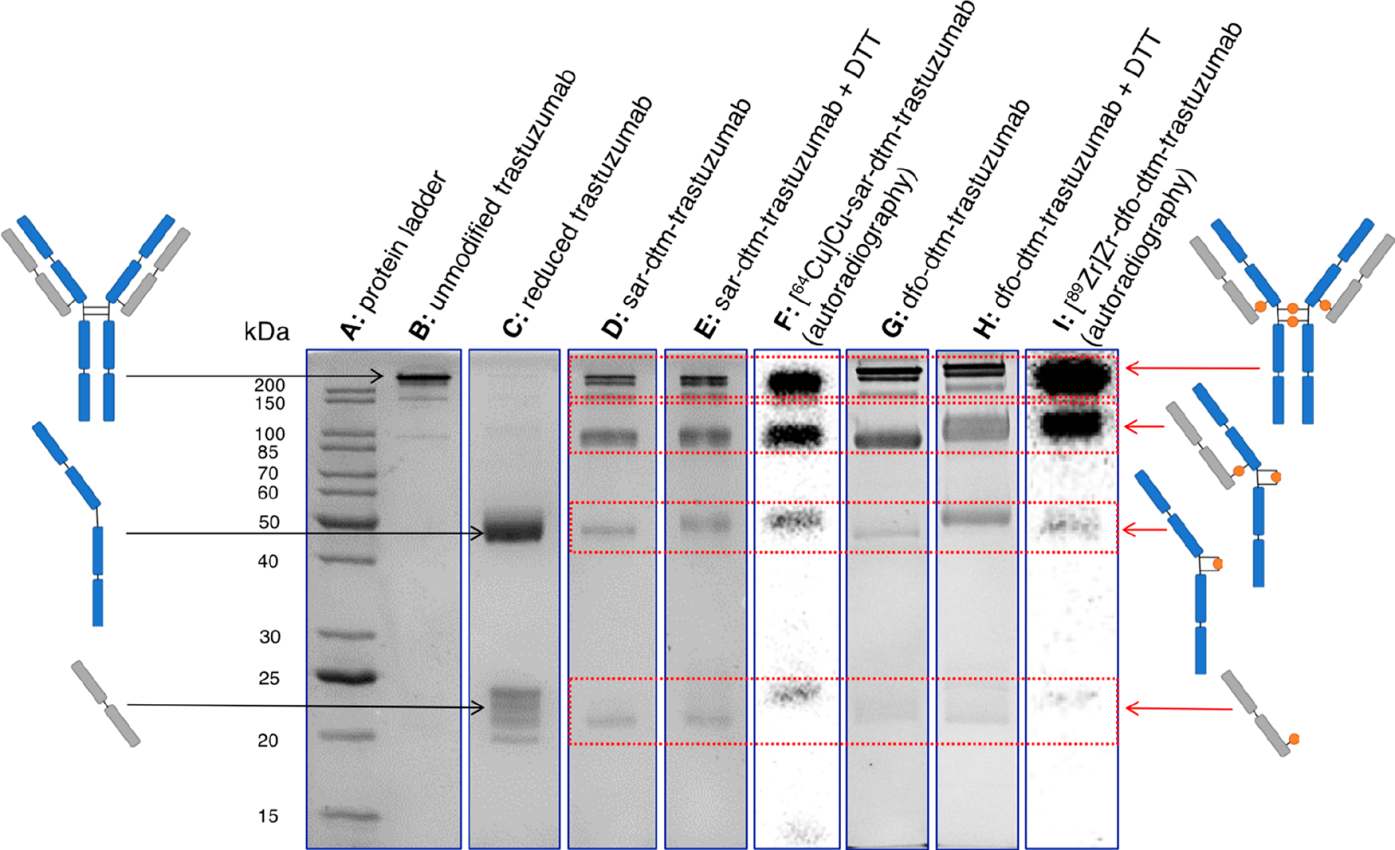
SDS-PAGE of chelator–dtm–trastuzumab conjugates. The full gels are included in Figures S3–S5.

As expected, the SDS-PAGE of TCEP-reduced trastuzumab showed the presence of separate heavy and light chains under the denaturing analysis conditions (Figure 1, lane C). Following dithiol rebridging reactions with chelator–dbm (for example sar–dbm, Figure 1, lane D, and dfo–dbm, Figure 1, lane G) and subsequent hydrolysis, the two major products observed corresponded to the “full antibody”, in which the maleamic acid groups bridge *inter* chain dithiols at the hinge region, and the “half antibody”, in which the maleamic acid groups bridge *intra* chain dithiols at the hinge region. The “full antibody” rebridging pattern mirrors that of the native trastuzumab antibody. The “half antibody” corresponds to a covalently linked single heavy chain + single light chain and is typical of this type of reaction.^31,34^ It arises as a result of “disulfide scrambling” and is only observed by harsh denaturing analysis. Less intense bands, corresponding to antibody heavy chains and light chains that remained “unbridged”, were also discernible, and these were more prominent for sar–dtm–trastuzumab than dfo–dtm–trastuzumab.

The same conjugation products were also analyzed using *reducing* SDS-PAGE conditions: dithiothreitol (DTT) was incubated with the antibody conjugate prior to running the SDS-PAGE. The same two “full antibody” and “half antibody bands” were observed (Figure 1, lanes E and H), indicating that (i) the conjugation and subsequent hydrolysis reactions to yield maleamic acid-conjugated motifs were complete, and (ii) the rebridged products were stable in the presence of excess thiols, consistent with prior reports detailing the high stability of such conjugates.^31,34^

The bioconjugates sar–dtm–trastuzumab and dfo–dtm–trastuzumab were also characterized by ESI-HRMS, which showed that both sar–dtm–trastuzumab and dfo–dtm–trastuzumab contain two–four copies of each chelator. The most intense signals in the deconvoluted ESI-HRMS of trastuzumab conjugates (Tables 1, S2, Figures S6, S7) corresponded to “half antibody”. Signals corresponding to “full antibody” were weak, which is a common feature of such immunoconjugates as the smaller fragments are always detected with greater relative intensity. Additionally, all deconvoluted “half antibody” and “full antibody” signals were significantly broader than that observed for similarly prepared and characterized alkyne–trastuzumab immunoconjugates.^34,42^ We attribute this peak broadening to sar–dtm–trastuzumab and dfo–dtm–trastuzumab readily complexing trace metal ions: we have previously observed trace metal binding to chelator conjugates in our mass spectrometric analyses.^43,44^

**Table 1:** ESI-MS Data for Chelator–Trastuzumab Conjugates

half antibody conjugate^a^	*m*/*z* (observed)	*m*/*z* (calculated)
**sar–dtm–trastuzumab**		
HL + 1 sar	73125	73116
HL + 2 sar	73647	73639
**dfo–dtm–trastuzumab**		
HL + 1 dfo		
HL + 2 dfo	74015	74017

a Abbreviation: HL = heavy chain + light chain (i.e., half antibody).

The combination of SDS-PAGE and ESI-HRMS data indicates that the new immunoconjugates consist of a heterogeneous mixture, comprised of predominantly “full antibody” and “half antibody” constructs (with low amounts of conjugate in which some heavy and light chains remain “unbridged”), and contain two–four chelators per antibody. This heterogeneity, and in particular the presence of both “full antibody” and “half antibody” constructs, is unlikely to significantly modify the immunoconjugates’ affinity for the target HER2 receptor or their biodistribution *in vivo*: we have previously shown that mixtures containing similar “disulfide-scrambled” trastuzumab species exhibit comparable antigen-binding and Fc-binding properties to native trastuzumab.^42^

**Figure 2:**
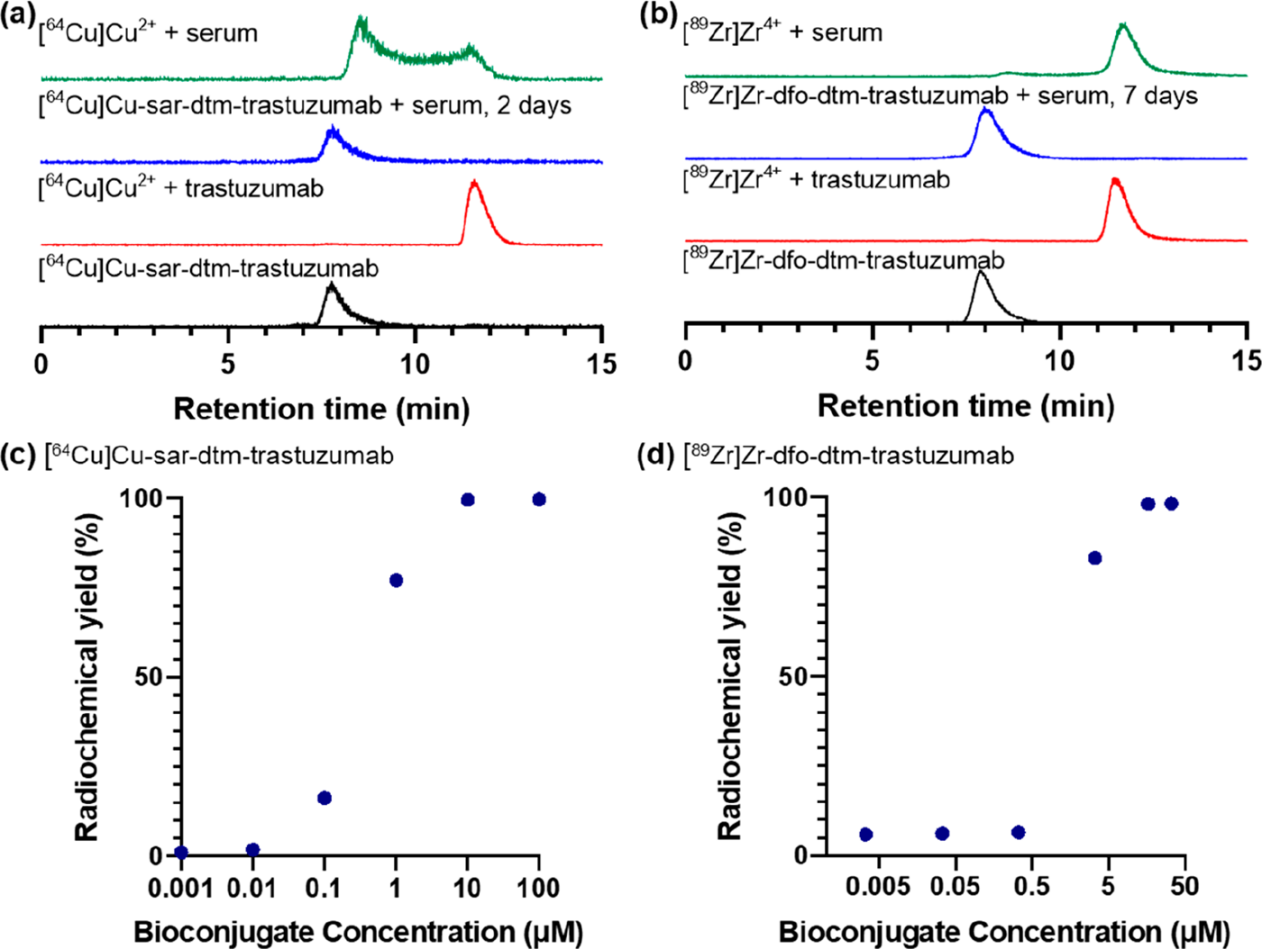
(a) SE-HPLC radiochromatograms of [^64^Cu]Cu-sar–dtm–trastuzumab (black), [^64^Cu]Cu^2+^ with unmodified trastuzumab (red), [^64^Cu]Cu-sar–dtm–trastuzumab incubated in serum for 2 days (blue), and [^64^Cu]Cu^2+^ incubated in serum (green). (b) SEC-HPLC radiochromatograms of [^89^Zr]Zr-dfo–dtm–trastuzumab (black), [^89^Zr]Zr^4+^ with unmodified trastuzumab (red), [^89^Zr]Zr-dfo–dtm–trastuzumab incubated in serum for 7 days (blue), and [^89^Zr]Zr^4+^ incubated in serum (green). (c) Radiochemical yields for the reaction of [^64^Cu]Cu^2+^ with sar–dtm–trastuzumab at different concentrations (100 μM, 10 μM, 1 μM, 100 nM, 10 nM, and 1 nM) of immunoconjugate at ambient temperature for 5 min. (d) Radiochemical yields for the reaction of [^89^Zr]Zr^4+^ with dfo–dtm–trastuzumab at different concentrations (33 μM, 16.7 μM, 3.3 μM, 333 nM, 33 nM, and 3 nM) of immunoconjugate at ambient temperature for 10 min.

The immunoconjugate sar–dtm–trastuzumab was radiolabeled with [^64^Cu]Cu^2+^ at ambient temperature and pH 7 in 5 min, by addition of a solution of [^64^Cu]Cu^2+^ (5 MBq, 30 μL, 0.1 M NH_4_OAc) to the immunoconjugate (9.43 μL, 100 μg, 0.1 M NH_4_OAc), and analyzed by size exclusion (SE) HPLC. In SE-HPLC, a PBS mobile phase containing ethylenediamine tetraacetate (EDTA) was used, and under these conditions, [^64^Cu]Cu-sar–dtm–trastuzumab eluted at 7.8 min (Figure 2a, bottom trace) and unreacted [^64^Cu]Cu^2+^ eluted at 11.6 min as [^64^Cu][Cu(EDTA)]^2–^. This reaction provided [^64^Cu]Cu-sar–dtm–trastuzumab in near quantitative radiochemical yield (>98%). Similarly, dfo–dtm–trastuzumab (10 μL, 72.5 μg, 0.1 M NH_4_OAc) was radiolabeled with [^89^Zr]Zr^4+^ (0.1 MBq, 5 μL, 0.2 M HEPES) at room temperature and pH 7 in 10 min, to yield [^89^Zr]Zr-dfo–dtm–trastuzumab in 99% radiochemical yield (Figure 2b, bottom trace). In contrast, when a sample of unmodified trastuzumab was reacted with either [^64^Cu]Cu^2+^ or [^89^Zr]Zr^4+^, <2% of radioactivity was associated with the antibody, with the majority of unreacted radiometal eluting at 11.6 min (Figure 2a,b).

[^64^Cu]Cu-sar–dtm–trastuzumab and [^89^Zr]Zr-dfo–dtm–trastuzumab were additionally analyzed by SDS-PAGE with bright field imaging and autoradiography: a radioactivity signal from radiolabeled immunoconjugates was coincident with stained protein bands (Figure 1, lanes F and I). For [^64^Cu]Cu-sar–dtm–trastuzumab, 41.1% of ^64^Cu signal was associated with “full antibody”, 33.4% was associated with “half antibody”, 14.5% was associated with single heavy chain fragments, and 10.6% was associated with single light chain fragments. For [^89^Zr]Zr-dfo–dtm–trastuzumab, 54.4% of ^89^Zr signal was associated with “full antibody”, 34.9% was associated with “half antibody”, 7.7% was associated with single heavy chain fragments, and 3.0% was associated with single light chain fragments. The comparatively lower amount of radiolabeled single heavy chain and single light chain fragments for [^89^Zr]Zr-dfo–dtm–trastuzumab relative to [^64^Cu]Cu-sar–dtm–trastuzumab suggested that dithiol rebridging was more efficient for dfo–dbm than sar–dbm.

To assess the serum stability of [^64^Cu]Cu-sar–dtm–trastuzumab and [^89^Zr]Zr-dfo–dtm–trastuzumab *ex vivo*, the radiolabeled immunoconjugates were incubated in human serum at 37 °C. [^64^Cu]Cu-sar–dtm–trastuzumab and [^89^Zr]Zr-dfo–dtm–trastuzumab both exhibited high stability in the presence of serum proteins: SE-HPLC radiochromatographic analysis indicated that >99% of [^64^Cu]Cu-sar–dtm–trastuzumab remained intact after 2 days incubation in serum and >99% of [^89^Zr]Zr-dfo–dtm–trastuzumab remained intact after 7 days incubation in serum (Figure 2 a,b, S8, S9). In contrast, when [^64^Cu]Cu^2+^ was incubated in serum, SE-HPLC analysis showed that the majority of [^64^Cu]Cu^2+^ was complexed by a serum protein (retention time of 8.5 min) or low molecular weight species (retention time of 11.6 min). When [^89^Zr]Zr^4+^ was incubated in serum, the majority of radioactivity was associated with low molecular weight species (retention time of 11.8 min).

For preparations of radiopharmaceuticals based on chelator–protein bioconjugates, it is important that the amount of the precursor bioconjugate is relatively low. Typically, unlabeled immunoconjugate is not separated from the radiolabeled conjugate prior to *in vivo* administration, and high concentrations of unlabeled bioconjugate lead to *in vivo* receptor “blocking”, compromising PET image contrast. Furthermore, the ability to radiolabel in near quantitative radiochemical yields obviates purification steps. To assess the ability of sar–dtm–trastuzumab or dfo–dtm–trastuzumab to complex [^64^Cu]Cu^2+^ or [^89^Zr]Zr^4+^ respectively, solutions containing radiometal were added to increasingly dilute solutions of immunoconjugate (Figure 2c,d). Near quantitative radiochemical yields (>99%) of [^64^Cu]Cu-sar–dtm–trastuzumab were achieved at immunoconjugate concentrations as low as 10 μM, pH 7 and ambient temperature, with only 5 min reaction time. At a lower sar–dtm–trastuzumab concentration of 1 μM, a high radiochemical yield of 77% was still obtained. Near quantitative radiochemical yields (>99%) of [^89^Zr]Zr-dfo–dtm–trastuzumab were achieved at 16 μM of dfo–dtm–trastuzumab at pH 7, ambient temperature and only 10 min reaction time. At a lower concentration of 3 μM, a relatively high radiochemical yield of 83% was still observed.

## *In Vitro* and *in Vivo* Characterization of [^64^Cu]Cu-sar–dtm–Trastuzumab

The binding of [^64^Cu]Cu-sar–dtm–trastuzumab to its target receptor, HER2, was evaluated in HER2-positive human ovarian cancer cells (SKOV3 cells). [^64^Cu]Cu-sar–dtm–trastuzumab retained specificity for HER2 receptor: the radiotracer showed uptake in SKOV3 cells, with uptake inhibited by increasing concentrations of unmodified trastuzumab (Figure S10).

**Figure 3:**
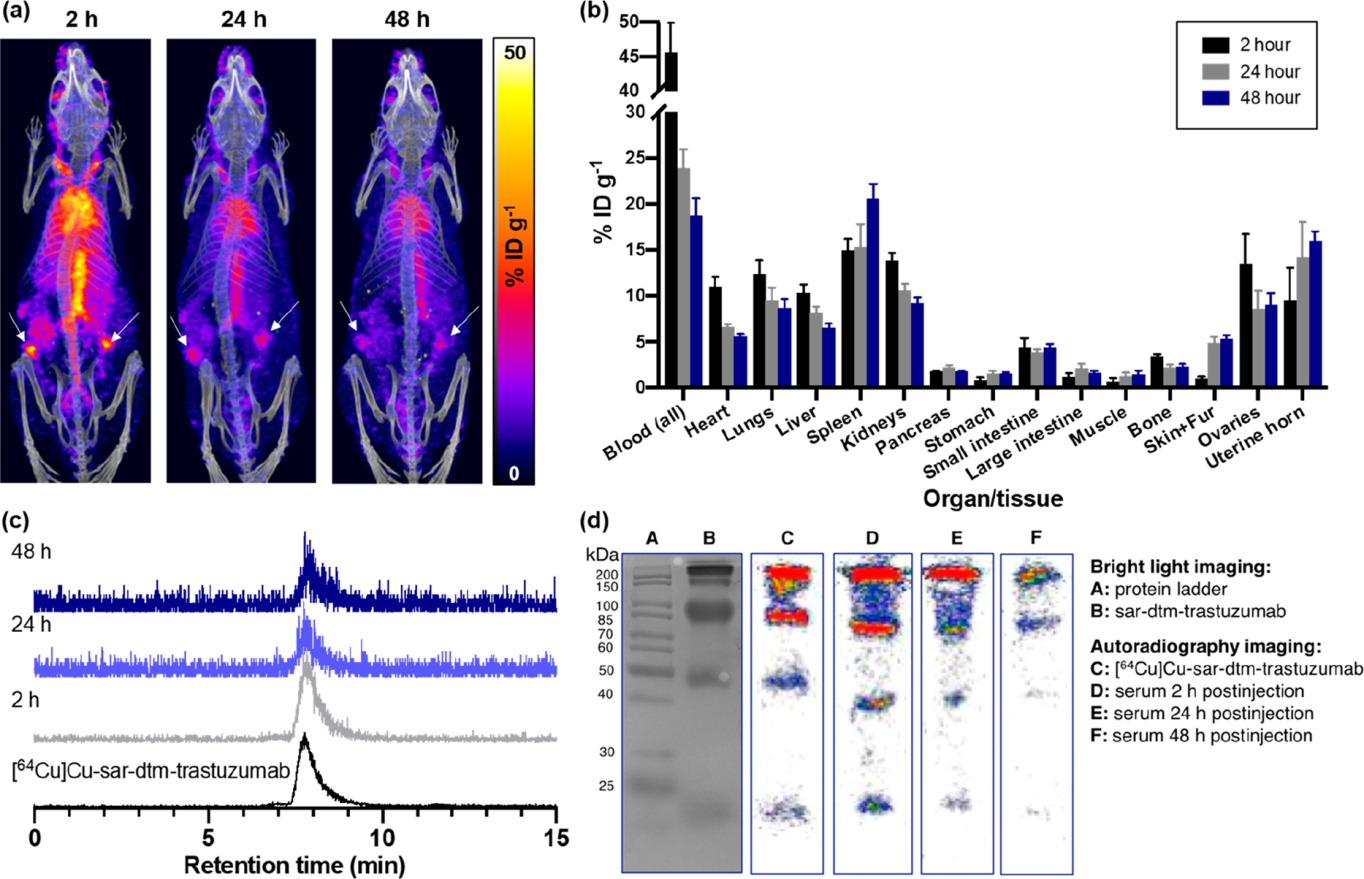
(a) PET/CT maximum intensity projections of a healthy female NOD scid gamma mouse administered [^64^Cu]Cu-sar–dtm–trastuzumab, at 2, 24, and 48 h postinjection. White arrows indicate ovaries that are positive for HER2. (b) *Ex vivo* biodistribution of [^64^Cu]Cu-sar–dtm–trastuzumab (*n* = 4 mice per time point). Error bars correspond to standard deviation. (c) SE-HPLC analysis of *ex vivo* serum samples shows a single radioactive signal in each chromatogram, with the retention time of each signal corresponding to [^64^Cu]Cu-sar–dtm–trastuzumab. (d) SDS-PAGE of *ex vivo* serum samples (bright light and autoradiography imaging) shows radioactive bands corresponding to [^64^Cu]Cu-sar–dtm–trastuzumab. The full gels are included in Figures S12, S13.

We have previously assessed *in vivo* stability of radiometal–chelator complexes by quantifying the biodistribution of their IgG-based immunoconjugates in healthy mice over an extended period of time.^3,19^ In healthy mice, antibodies such as trastuzumab have long blood half-lives in the absence of diseased tissues/tumors that are positive for the target receptor. Here, *in vivo* studies in healthy animals aimed to assess the biodistribution and stability of [^64^Cu]Cu-sar–dtm–trastuzumab *in vivo*. [^64^Cu]Cu-sar–dtm–trastuzumab (1.0–2.5 MBq) was administered intravenously (via tail vein) to NOD scid gamma female mice. At 2 h, 1 day, and 2 days postinjection, PET/CT images were acquired, mice were culled, and organs and serum samples were collected for *ex vivo* analysis (Figures 3, S11).

PET/CT imaging and *ex vivo* biodistribution (Figures 3a,b, S11) showed that significant ^64^Cu radioactivity remained in blood circulation over 1–2 days postinjection, although as typically observed, this activity decreased from 45.6 ± 4.3%ID (percentage injected dose) g^–1^ at 2 h postinjection to 18.7 ± 1.9%ID g^–1^ at 2 days postinjection. Importantly, ^64^Cu radioactivity in the liver did not increase from 2 h to 2 days postinjection. Prior studies^3,45^ have shown that loss of [^64^Cu]Cu from a bioconjugate (likely via transchelation to endogenous proteins including albumin in blood^46^ and superoxide dismutase^47^ and ceruloplasmin^48^ in the liver) results in increased accumulation of [^64^Cu]Cu in the liver over time. Our biodistribution data is consistent with prior reports that detail exceptional stability of [^64^Cu][Cu(sar)]^2+^ conjugates (both peptide and protein conjugates) *in vivo*.^3,45^

Radioactivity uptake and retention in healthy HER2-expressing tissue also indicated that [^64^Cu]Cu-sar–dtm–trastuzumab retains affinity for its target HER2 receptor (Figures 3a,b, S11). Notably, skin, uterine horn, and ovaries–healthy tissues that are known to express HER2^49,50^ −showed either increased uptake or persistent retention of ^64^Cu radioactivity over the course of the study. For example, radioactivity uptake in skin increased from 1.0 ± 0.2%ID g^–1^ at 2 h postinjection to 4.9 ± 0.7%ID g^–1^ at 1 day postinjection (*p* = 3.1 × 10^–5^); at 2 days postinjection, skin samples showed retention of ^64^Cu radioactivity (5.3 ± 0.4%ID g^–1^).

Serum samples were obtained from mice administered [^64^Cu]Cu-sar–dtm–trastuzumab at 2 h, 1 day, and 2 days postinjection and analyzed by SE-HPLC and SDS-PAGE. In each SE-HPLC chromatogram, only a single signal was observed, with a retention time matching that of [^64^Cu]Cu-sar–dtm–trastuzumab (Figure 3c). Serum samples were also subjected to SDS-PAGE separation, followed by autoradiography imaging, which revealed radioactive bands (Figure 3d, lanes D–F) that corresponded to denatured fragments of both unlabeled sar–dtm–trastuzumab (Figure 3d, lane B) and [^64^Cu]Cu-sar–dtm–trastuzumab (Figure 3d, lane C). Significantly, neither SE-HPLC nor SDS-PAGE analyses revealed any radiolabeled species with molecular weights corresponding to serum albumin (with a molecular weight of 66.5 kDa), which is known to (i) react with maleimide derivatives released *in vivo* as a result of intramolecular retro-Michael reactions of bioconjugates, resulting in transfer of maleimide groups and their cargo from bioconjugates to albumin,^21−24^ and additionally, (ii) compete with [^64^Cu]Cu-radiolabeled chelator conjugates of peptides and proteins for [^64^Cu]Cu^2+^ binding, resulting in release of [^64^Cu]Cu from chelator conjugates.^46^ In short, our experiments evidence the high stability of [^64^Cu]Cu-sar–dtm–trastuzumab *in vivo*.

### Concluding Remarks: Usability of Chelators Containing Dibromomaleimide Groups for Site-Specific Radiolabeling of an IgG Antibody

The majority of chelator–antibody conjugates are prepared from chelators containing reactive electrophilic groups that react with solvent accessible primary amines of lysine side chains. While these conjugation protocols are very simple to implement (even for researchers inexperienced in protein chemistry), such methods mostly lead to heterogeneous mixtures of chelator–protein conjugates that can exhibit suboptimal pharmacokinetics and decreased affinity for target receptors.^6−8^ Immunoconjugates containing conventional maleimide groups can enable site-specific attachment to reduced thiol groups of disulfide bonds of antibody derivatives, but the resulting conjugates undergo retro-Michael reactions in the biological milieu, resulting in loss of radioactive cargo.^21−24^ Many new chemical innovations in site-specific modification of antibodies have been adapted to stably incorporate chelators into antibodies and their derivatives;^9−14^ however, these methods involve enzymatic reactions, engineering of specific peptide sequences into the antibody and/or multistep procedures, and are therefore not simple to implement and tend to require extensive optimization for them to work reliably.

Dibromomaleimide chemistry is superbly suited to simple, stable, and site-specific incorporation of radionuclides into antibodies for receptor-targeted molecular imaging. We have prepared dibromomaleimide derivatives of (i) a dfo chelator, commonly used for [^89^Zr]Zr^4+^ radiolabeling of antibodies, and (ii) a sarcophagine chelator, which is considered state-of-the-art for [^64^Cu]Cu^2+^ radiolabeling of proteins and peptides, and successfully incorporated both new bifunctional chelators into the HER2-targeted IgG antibody, trastuzumab. These are the first examples of radiometal-labeled chelator–antibody conjugates that have been prepared using dibromomaleimide technology, enabling site-specific attachment of two different chelators to the IgG trastuzumab antibody. Importantly, the methods we describe are simple to implement and produce chelator–antibody conjugates that can be readily radiolabeled with the PET radiometals [^89^Zr]Zr^4+^ and [^64^Cu]Cu^2+^ in near-quantitative radiochemical yields at ambient temperature. We have evidenced the high stability of [^64^Cu]Cu-sar–dtm–trastuzumab both in serum and *in vivo*: it is stable to both demetalation of [^64^Cu]Cu, consistent with the known high stability of [^64^Cu][Cu(sar)]^2+^ complexes, and transfer of the [^64^Cu][Cu(sar)]^2+^ to endogenous thiols, consistent with the reported stability of dithiomaleamic acid groups. Our future efforts will compare the biological properties of these site-specifically radiolabeled immunoconjugates with radiolabeled immunoconjugates modified using either conventional maleimide conjugation chemistry or non-site-specific (stochastic) conjugation chemistry.

## Supporting Information Available

Experimental descriptions, full SDS-PAGE data, ESI-MS, NMR, HPLC chromatograms, PET/CT image analysis, cell binding study data (PDF)
