# Supplementary material for: New Bifunctional Chelators Incorporating Dibromomaleimide Groups for Radiolabeling of Antibodies with Positron Emission Tomography Imaging Radioisotopes
Source: Bioconjug Chem. 2021 Mar 16;32(7):1214–22. doi: 10.1021/acs.bioconjchem.0c00710 (PMC8299457; doi:10.1021/acs.bioconjchem.0c00710)
Supplement: Supplementary file 1 — bc0c00710_si_001.pdf [file bc0c00710_si_001.pdf]

New bifunctional chelators incorporating dibromomaleimide groups for radiolabeling of antibodies with PET imaging radioisotopes

Supporting Information

Matthew Farleigh,[1] Truc Thuy Pham,[1] Zilin Yu,[1] Jana Kim,[1] Kavitha Sunassee,[1] George Firth,[1] Nafsika Forte,[2] Vijay Chudasama,[2] James R. Baker,[2] Nicholas J. Long,[3] Charlotte Rivas[1], Michelle T. Ma*[1]*

[1]School of Biomedical Engineering and Imaging Sciences, King's College London, St Thomas' Hospital, London, SE1 7EH, UK

[2]Department of Chemistry, University College London, 20 Gordon Street, London, WC1H 0AJ, UK

[3]Department of Chemistry, Imperial College London, Molecular Sciences Research Hub, London, W12 0BZ, UK

*charlotte.rivas@kcl.ac.uk, michelle.ma@kcl.ac.uk

Table of Contents

Materials and instrumentation 3

Bifunctional chelator synthesis 3

Preparation of sar-dtm-trastuzumab and dfo-dtm-trastuzumab immunoconjugates 4

Radiolabelling of sar-dtm-trastuzumab with [^{64}Cu]Cu^{2+} 6

Radiolabelling of dfo-dtm-trastuzumab with [^{89}Zr]Zr^{4+} 7

Serum stability and target receptor affinity of [^{64}Cu]Cu-sar-dtm-trastuzumab and [^{89}Zr]Zr-dfo-dtm-trastuzumab 7

In vivo assessment of [^{64}Cu]Cu-sar-dtm-trastuzumab 8

Figures 10

- Figure S1. SDS-PAGE of conjugation reaction between reduced trastuzumab and sar-dbm 10
- Figure S2. SDS-PAGE of conjugation reaction between reduced trastuzumab and dfo-dbm 11
- Figure S3. SDS-PAGE of [^{64}Cu]Cu-sar-dtm-trastuzumab. 11
- Figure S4. SDS-PAGE of [^{89}Zr]Zr-dfo-dtm-trastuzumab. 12
- Figure S5. SDS-PAGE of sar-dtm-trastuzumab and dfo-dtm-trastuzumab 12
- Figure S6. High resolution ESI deconvoluted mass spectrum of sar-dtm-trastuzumab 13
- Figure S7. High resolution ESI deconvoluted mass spectrum of dfo-dtm-trastuzumab 14

Figure S8. Size exclusion HPLC chromatograms of [^{64}Cu]Cu-sar-dtm-trastuzumab incubated with serum 15

Figure S9. Size exclusion HPLC chromatograms of [^{89}Zr]Zr-dfo-dtm-trastuzumab incubated with serum 15

Figure S10. Cell binding study of [^{64}Cu]Cu-sar-dtm-trastuzumab with HER2-positive SKOV3 cells. 15

Figure S11. Biodistribution of [^{64}Cu]Cu-sar-dtm-trastuzumab in healthy female NOD scid gamma mice determined from PET/CT imaging 16

Figure S12. SDS-PAGE of all *ex vivo* serum samples 16

Figure S13. SDS-PAGE of *ex vivo* serum samples 17

Figure S14. 1H NMR spectrum for $(NH_2)_2$sar 17

Figure S15. ^{1}H NMR spectrum for $[Mg(NH_2)_2sar](CF_3SO_3)_2$ 18

Figure S16. ^{1}H NMR spectrum for sar-dbm 18

Figure S17. ^{1}H NMR spectrum for dfo-dbm. 19

Figure S18. ^{13}C NMR spectrum for sar-dbm 19

Figure S19. ^{13}C NMR spectrum for dfo-dbm 20

Figure S20. High-resolution ESI-mass spectrum for sar-dbm 21

Figure S21. High-resolution ESI-mass spectrum for dfo-dbm 22

Figure S22. Analytical C18 reverse-phase HPLC chromatograms of sar-dbm and dfo-dbm 23

Figure S23. LC-MS analysis of a crude reaction mixture containing sar-dbm and sar-dbm$_2$ 23

Figure S24. ^{1}H NMR spectra for sar-dbm$_2$ 23

Figure S25. High-resolution ESI-mass spectrum for sar-dbm$_2$ 23

References 27

Materials and instrumentation

Chemicals and reagents were obtained from Sigma-Aldrich (Dorset, UK) unless otherwise specified. The highest available purity (lowest metal ion-containing) chemicals were used. 18.2 MΩ water was used to prepare all solutions. PD-10 size exclusion columns were purchased from GE Healthcare UK Ltd. (Buckinghamshire, UK). Trastuzumab was obtained as the biosimilar Herzuma in solution (21 mg/mL) from the Pharmacy Department at Guy's and St. Thomas' NHS Trust, London. Fresh human serum was obtained from a healthy volunteer. Preparative high-performance liquid chromatography (HPLC) purification was carried out using an Agilent PrepStar LC system with in-line UV. NMR spectra were acquired on either a Bruker 400 MHz spectrometer, or a Bruker 700 MHz (Bruker UK Limited, Coventry, UK). NMR spectra were referenced to residual solvent signals, or in the case where deuterium oxide was the solvent in ^{13}C NMR spectra, a trifluoroacetic acid reference was used. Analytical reverse phase HPLC was carried out using an Agilent 1260 Infinity II equipped with an Eclipse XDB-C18 column (4.6 × 150 mm, 5 µm) with a 1 mL min^{-1} flow rate and UV spectroscopic detection at 220 nm, 254 nm, and 330 nm, and a gradient mobile phase with a flow rate of 1 mL min^{-1}. (Gradient: A = 0.1% FA in water, B = 0.1 % FA in acetonitrile. 0-2 min 5 % B in A, 2-11 min 5 %→95 % B in A, 12-12.10 min 95 %→5 % B in A, 12.10-15 min 5 % B in A.) Instant thin layer chromatography strips (ITLC-SG) were obtained from Varian Medical Systems UK, Ltd. (Crawley, UK), and ITLC strips were visualised using a Lab Logic B-MS-1000 plate reader with a B-FC-2000P detector equipped with a B-FC-3600 photomultiplier. Analytical size exclusion radioHPLC traces were acquired using an Agilent 1200 Series HPLC system with an in-line radioactivity detector (Ray Test Gabi with a Ray Test Na-I 1x1" Std. detector). A BioSep™ SEC-s2000 LC Column (5 µm, 145 Å, 300 x 7.8 mm) was used with a mobile phase of PBS and sodium ethylenediamine tetraacetate (2 mM) at a flow rate of 1 mL min^{-1}.

Bifunctional chelator synthesis

[Mg(NH$_2$)$_2$sar](CF$_3$SO$_3$)$_2$

$(NH_2)_2$sar was synthesised following literature methods.[1,2] Preparation of $[Mg(NH_2)_2sar](CF_3SO_3)_2$ was adapted from the literature.[3] $(NH_2)_2$sar (100 mg, 318 µmol) and $Mg(CF_3SO_3)_2$ (103 mg, 318 µmol) were dissolved in dry acetonitrile (5 mL) and refluxed for 30 min, then cooled to room temperature. The solution was then evaporated to dryness to yield a white solid. $[Mg(NH_2)_2sar](CF_3SO_3)_2$: 176 mg, 277 µmol, 87 %. ^1H NMR (400 MHz, D_2O) δ 3.61 (m, 6H), 3.15 (m, 12H), 2.68 (m, 6H), 2.42 (m, 6H).

sar-dbm

$[Mg(NH_2)_2sar](CF_3SO_3)_2$ (250 mg, 393 µmol, 1 eq.) was dried in vacuo; separately 3,4-dibromo-maleimide-N-hexanoic acid[4] (446 mg, 1.214 mmol, 3 eq.) and EEDQ (296 mg, 1.197 mmol, 3 eq) were also dried in vacuo. 3,4-dibromo-maleimide-N-hexanoic acid and EEDQ were added to dry acetonitrile (20 mL) and stirred for 1 hour at ambient temperature. $[Mg(NH_2)_2sar](CF_3SO_3)_2$ was added and the reaction was stirred for 3 hours at ambient temperature. Glacial acetic acid (12.5 mL) was then added and the solution evaporated to dryness. Water (10 mL) was added to the dry residue, and the supernatant was separated from insoluble material. The supernatant was lyophilised to give a white powder, containing the desired product. The crude product was purified by preparative reverse phase HPLC (Agilent PrepHT XDB-C18) using mobile phase A (water with 0.1 % trifluoroacetic acid) and mobile phase B (acetonitrile with 0.1 % trifluoroacetic acid) with an eluting gradient (10 mL min^{-1}, 0-5 min 0 % B in A, 5-10 min 0 % → 20 % B in A, 10-40 min 20 % → 60 % B in A). The product was eluted in a single fraction (retention time 21.5 min) which was lyophilised to give an off-white powder (20.7 mg, 8 % yield). ^1H NMR (700 MHz, D_2O) δ 3.50 (s, 3H), 3.45 (t, J = 8.2 Hz, 2H), 3.15 (s, 12H), 3.00 (s, 12H), 2.28 (s, 3H), 2.11 (t, J = 8.2 Hz, 2H), 1.43 (m, 4H), 1.12 (m, 2H). ^{13}C NMR (176 MHz, D_2O) δ 178.55, 166.45, 129.33, 55.91, 54.92, 50.83, 50.45, 47.86, 46.14, 39.47, 35.94, 27.44, 25.55, 24.34. High resolution ESI-MS +ve mode, $[C_{24}H_{44}Br_2N_9O_3]^+$ m/z = 666.1915 (observed), 666.1936 (expected). Analytical HPLC retention time, 5.92 min (see materials and instrumentation for HPLC specifications). See Figures S16, S18, S20 and S22.

dfo-dbm

3,4-Dibromo-maleimide-1-acetic acid[4] (100 mg, 321 µmol) and EEDQ (279 mg, 1.13 mmol) were dissolved in dimethylsulfoxide (DMSO) (4 mL) with stirring. Desferrioxamine (dfo) mesylate (627 mg, 963 µmol) was added, and the reaction was stirred for 3 h. The reaction was then quenched by the addition of TFA (40 µL). The crude product was purified by preparative reverse phase HPLC (Agilent PrepHT XDB-C18) using mobile phase A (water with 0.1 % trifluoroacetic acid) and mobile phase B (acetonitrile with 0.1 % trifluoroacetic acid) with an eluting gradient (10 mL min^{-1}, 0-10 min 5 % B in A, 10-60 min 5 % → 35 % B in A). The product was obtained in one fraction which was lyophilised to give a white powder, 61.2 mg (retention time 43.5 min, 22 % yield). ^{1}H NMR (400 MHz, DMSO) δ 9.57 (s, 3H), 8.13 (t, *J* = 5.5 Hz, 1H), 7.74 (t, *J* = 5.2 Hz, 2H), 4.08 (s, 2H), 3.45 (t, *J* = 7.0 Hz, 6H), 3.07 – 2.96 (m, 6H), 2.57 (t, *J* = 7.1 Hz, 4H), 2.27 (t, *J* = 7.3 Hz, 4H), 1.96 (s, 3H), 1.55 – 1.45 (m, 6H), 1.43 – 1.32 (m, 6H), 1.28 – 1.16 (m, 6H). ^{13}C NMR (101 MHz, DMSO) δ 171.95, 171.25, 170.12, 165.26, 163.91, 129.50, 47.07, 41.41, 39.52, 38.38, 29.90, 28.77, 27.52, 25.99, 23.46, 20.28. High-resolution ESI-MS +ve mode, $[C_{31}H_{50}Br_2N_7O_{11}]^+$ m/z = 856.1912 (observed), m/z = 856.1915 (expected). Analytical HPLC retention time, 7.95 min (see materials and instrumentation for HPLC specifications). See Figures S17, S19, S21 and S22.

Preparation of sar-dtm-trastuzumab and dfo-dtm-trastuzumab immunoconjugates

sar-dtm-trastuzumab

This method was modified from the literature.[5] Trastuzumab (5 mg) was dissolved in aqueous borate buffered saline (BBS, 1.5 mL) solution (50 mM sodium borate, 50 mM NaCl, and 5 mM ethylenediaminetetraacetic acid (EDTA), pH 8.5) to give an antibody concentration of 23 µM. To this antibody solution was added tris(2-carboxyethyl)phosphine solution (TCEP, 10 mM in BBS, 21 µL, 6 eq.) and warmed at 37 °C for 2 h. Then sar-dbm (10 mM in water, 28 µL, 8 eq.) was added to the reduced antibody solution where a colour change from a colourless to pale yellow solution was observed. This solution was then warmed at 37 °C for 48 h. The bioconjugate was loaded onto a PD-10 column (preconditioned with aqueous ammonium acetate (0.2 M) solution) and eluted with aqueous ammonium acetate (0.2 M) in 0.5 mL fractions. Fraction protein content was assessed using a NanoDrop ™ 2000 spectrophotometer. Fractions 6 and 7 were combined, and further purified and buffer exchanged into aqueous ammonium acetate (0.1 M) by six cycles of spin filtration (4000 rcf, 15 min) through a Vivaspin (50 kDa MWCO). The bioconjugate sar-dtm-trastuzumab (3.62 mg) was recovered in 200 µL solution at a concentration of 125 µM.

dfo-dtm-trastuzumab

This method is modified from the literature.[5] Trastuzumab (5 mg) was dissolved in borate buffered saline (BBS, 1.5 mL) solution (50 mM sodium borate, 50 mM NaCl, and 5 mM ethylenediaminetetraacetic acid (EDTA), pH 8.5) to give an antibody concentration of 23 µM. To this antibody solution was added tris(2-carboxyethyl)phosphine solution (TCEP, 10 mM in BBS, 21 µL, 6 eq.) and warmed at 37 °C for 2 h. Then DFO-dbm (10 mM in DMF, 28 µL, 8 eq.) was added to the reduced antibody solution where a colour change from a colourless to pale yellow solution was observed. This solution was then left for two hours at room temperature. The bioconjugate was loaded onto a PD-10 column (preconditioned with aqueous ammonium acetate (0.2 M) solution) and eluted with ammonium acetate (0.2 M) in 0.5 mL fractions. Fraction protein content was assessed using a NanoDrop ™ 2000 spectrophotometer. Fractions 6 and 7 were combined, and further purified and buffer exchanged into aqueous ammonium acetate (0.1 M) by six cycles of spin filtration (4000 rcf, 15 min) through a Vivaspin (50 kDa MWCO). The bioconjugate dfo-dtm-trastuzumab (2.36 mg) was recovered in 200 µL solution at a concentration of 81 µM.

SDS-PAGE analysis of trastuzumab conjugates

These methods are adapted from the literature.[5] A sample of immunoconjugate (3 µL) was diluted with water (10 µL), followed by addition of SDS-PAGE loading buffer (2 µL). This solution was mixed thoroughly and then heated to 65 °C for 5 min to denature the protein. The sample was then loaded onto the gel (12 % acrylamide with 4% stacking gel), along with a molecular weight marker, a sample of trastuzumab (prepared in the same way), and a sample of reduced trastuzumab (also prepared in the same way). The stability of the bioconjugates in the presence of a large excess of thiol was also assessed by preparing samples in the same fashion as described above, with the addition inclusion of dithiothreitol (DTT) (10 mM in water, 1 µL, ≥100 eq.) prior to denaturing. Separation of protein components was achieved by applying a constant voltage to the gels (35 mA, 35 min) in SDS running buffer. Gels were stained with Coomassie blue, destained, and then bright light imaged (Invitrogen, iBright FL1000 , Thermo Fisher Scientific).

ESI-MS analysis of bioconjugates

Molecular masses were measured using an Agilent 6510 QTOF LC-MS system (Agilent, UK). An Agilent 1200 HPLC system was equipped with an Agilent PLRP-S, 1000A, 8 µM, 150 mm x 2.1 mm column. Bioconjugates were buffer exchanged to ammonium acetate (0.2 M, pH 6.9) using a Vivaspin (50 kDa MWCO) to a final concentration of 6.5 µM. To 100 µL of this bioconjugate solution was added PNGase F (1.0 µL, New England Biolabs) at 37 °C for 16 h. After this time, the sample was diluted to 1.8 µM with ammonium acetate buffer (0.2 M, pH 6.9). 10 µL of bioconjugate sample (1.8 µM) was injected on the column using mobile phase A (water-0.1% formic acid) and B (acetonitrile-0.1% formic acid) with an eluting gradient (as shown in Table S1) at a flow rate of 300 µl/min. The oven temperature was maintained at 60 °C. An Agilent 6510 QTOF mass spectrometer was operated in a positive polarity mode, coupled with an ESI ion source. The ion source parameters were set up with a VCap of 3500V, a gas temperature at 350 °C, a dry gas flow rate at 10 L/min and a nebulizer of 30 psig. MS ToF was acquired under conditions of a fragmentor at 350 V, a skimmer at 65 V and an acquisition rate at 0.5 spectra/s in a profile mode, within a scan range between 700 and 5000 m/z. The data was then analysed by deconvoluting to a zero charge mass spectrum using a maximum entropy deconvolution algorithm.

Table S1. LC-MS mobile phase A/ B gradient elution.

Time (min)	Solvent A (%)	Solvent B (%)
0.0	85	15
1.0	85	15
1.50	68	32
2.0	68	32
7.0	50	50
9.0	5	95
10.0	5	95
12.0	85	15
15.0	85	15

Table S2: ESI-MS data for trastuzumab and chelator-trastuzumab conjugates

Antibody species	m/z (observed)	m/z (calculated)
**trastuzumab**		
HHLL	145186	
**sar-dtm-trastuzumab**		
L + 1 sar	23970	23963
HL + 1 sar	73126	73116
HL + 2 sar	73646	73639
HHLL + 1 sar	145694 (weak)	145709
HHLL + 2 sar	146236	146232
HHLL + 3 sar	146746	146755
HHLL + 4 sar	147277	147278
**DFO-dtm-trastuzumab**		
HL + 1 DFO	73307	73305
HL + 2 DFO	74015	74017
HHLL + 2 DFO	146604	146610
HHLL + 3 DFO	147324	147322
HHLL + 4 DFO	148032	148034

Radiolabelling of sar-dtm-trastuzumab with $[^{64}Cu]Cu^{2+}$

$[^{64}Cu]Cu^{2+}$ production and purification

A concave high purity gold target was electroplated with $[^{64}Ni]Ni$ (95.6 % isotopically enriched), rinsed with water and then dried.[6] This target was then irradiated with protons on a CTI RDS 112 cyclotron (11 MeV, 1 cm beam diameter) with a beam current of 30 µA, for 8 h to induce the $^{64}Ni(p,n)^{64}Cu$ reaction. HCl (9 M, 150 µL) was added to the irradiated surface and then evaporated at 95 °C. This was repeated three times. HCl (9 M, 300 µL) was added to the irradiated surface and then transferred to an anion exchange column (AG® 1-X8 Anion Exchange Resin, pre-rinsed with water and HCl (6 M)). This step was repeated three times to give a total of 900 µL added to the column. The column was then eluted sequentially under gravity with 6 M HCl (5 mL) to remove excess $[^{64}Ni]Ni^{2+}$; followed by 4 M HCl (5 mL), to remove any radio-cobalt impurities; 0.1 M HCl (10 mL) was used to elute $[^{64}Cu]Cu^{2+}$. Twenty fractions (1 mL each) were collected in total. Fractions 14 and 15 contained the majority of the $[^{64}Cu]Cu^{2+}$ produced, with a radionuclidic purity greater than 99.9 %.

Radiolabelling of sar-dtm-trastuzumab with $[^{64}Cu]Cu^{2+}$

An aliquot from fraction 15 containing $[^{64}Cu]Cu^{2+}$ (56.5 MBq, 448 µL) was dried under a stream of N_2 at 100 °C, and then redissolved in aqueous ammonium acetate solution (0.1 M, 339 µL). A sample of $[^{64}Cu]Cu^{2+}$ from this solution (5 MBq, 30 µL) was then added to an aqueous solution of sar-dtm-trastuzumab (100 µg, in 9.4 µL of 0.1 M ammonium acetate). The sample was agitated, and left at ambient temperature for 5 min. Radiochemical yields were determined using ITLC-SG strips spotted with 1 µL of radiolabelling mixture, which were developed in a mobile phase of 0.1 M citrate buffer (pH 5.5). For $[^{64}Cu]$Cu-sar-dtm-trastuzumab: $R_f < 0.1$; for unreacted $[^{64}Cu]Cu^{2+}$: $R_f > 0.9$. Size exclusion HPLC (see materials and instrumentation) was also used to assess radiochemical yield. $[^{64}Cu]$Cu-sar-dtm-trastuzumab had a retention time of 7.75 min, and unreacted $[^{64}Cu]Cu^{2+}$ eluted at 11.58 min. Near quantitative radiochemical yields (> 98%) were achieved at a specific activity of 43.6 MBq mg^{-1}, corresponding to a molar activity of 6.33 GBq μmol^{-1}.

$[^{64}Cu]Cu^{2+}$ radiolabelling of sar-dtm-trastuzumab at varying concentrations of bioconjugate

An aliquot from fraction 15 containing $[^{64}Cu]Cu^{2+}$ (1 MBq) was dried under a stream of N_2 at 100 °C, and then redissolved in aqueous ammonium acetate solution (0.1 M, 20 µL) to give a 50 kBq μL^{-1} solution (pH 7). Sar-dtm-trastuzumab was diluted to 125 µM in ammonium acetate (0.1 M). To 8 µL of this bioconjugate solution was added 2 µL (100 kBq) of $[^{64}Cu]Cu^{2+}$ in ammonium acetate (0.1 M), agitated and then left at room temperature

for 5 min (this dilution gives a 100 µM solution). ITLC was used to assess radiochemical yield. This process was repeated at bioconjugate concentrations of 10 µM, 1 µM, 100 nM, 10 nM, and 1 nM.

Radiolabelling of sar-dtm-trastuzumab with [^{64}Cu]Cu^{2+} for in vivo assessment

Aliquots from fraction 14 (132 µL, 28 MBq) and fraction 15 (1 mL, 73.4 MBq) were combined and dried under a stream of N_2 at 100 °C, and then redissolved in aqueous ammonium acetate solution (0.1 M, 573 µL). A sample of [^{64}Cu]Cu^{2+} from this solution (225 µL, 32.7 MBq) was then added to sar-dtm-trastuzumab (3.21 mg, 330 µL, in 0.1 M ammonium acetate). The sample was agitated, and left at ambient temperature for 5 min. As above, ITLC and size exclusion HPLC were used to assess radiochemical yield. Near quantitative radiochemical yields (> 99%) were obtained at a specific activity of 10.19 MBq mg^{-1} corresponding to a molar activity of 1.48 GBq µmol^{-1}.

Radiolabelling of dfo-dtm-trastuzumab with [^{89}Zr]Zr^{4+}

Preparation of [^{89}Zr]Zr^{4+} in HEPES buffer

No-carrier-added [^{89}Zr]Zr^{4+} (radionuclidic purity >99.9%) produced at the BV Cyclotron, Amsterdam, was purchased from Perkin Elmer, and was received as [^{89}Zr][Zr(ox)$_4$] (ox = oxalate). Oxalate was removed from solution using reported methods.[7] Briefly, SEP-Pak light QMA cartridge was preconditioned with EtOH (5 mL), saline (0.9 %, 10 mL), and water (10 mL). [^{89}Zr][Zr(ox)$_4$] (ox = oxalate) was diluted with water to a total volume of 250 µL and then loaded onto the preconditioned purification cartridge. The cartridge was washed with water (10 mL) to remove oxalic acid. To elute [^{89}Zr]Zr^{4+}, a solution of aqueous HCl (0.5 mL, 1 M) was used: the first 150 µL was discarded, and the remaining 350 µL contained [^{89}Zr]Zr^{4+}, which was then dried under a stream of N_2 at 100 °C. This was then re-dissolved in aqueous HEPES buffer (0.2 M, pH 7).

Radiolabelling of dfo-dtm-trastuzumab with [^{89}Zr]Zr^{4+}

The dfo-dtm-trastuzumab bioconjugate was diluted to a concentration of 50 µM in ammonium acetate (0.1 M). To 10 µL of this solution was added 5 µL of [^{89}Zr]ZrCl$_4$ (100 kBq) in HEPES (0.2 M) to give a 33.3 µM solution of bioconjugate. This was agitated and left at room temperature for 10 min. Radiochemical yield was determined using ITLC-SG strips spotted with 1 µL of radiolabelling mixture, which were developed in a mobile phase of 0.1 M citrate buffer (pH 5.5). For [^{89}Zr]Zr-dfo-dtm-trastuzumab: $R_f < 0.1$; for unreacted [^{89}Zr]Zr^{4+}: $R_f > 0.9$. Size exclusion HPLC was also used to assess radiochemical purity. [^{89}Zr]Zr-dfo-dtm-trastuzumab had a retention time of 7.75 min, whilst unreacted [^{89}Zr]Zr^{4+} eluted at 11.58. Near quantitative (> 99%) radiochemical yields were achieved.

Radiolabelling of dfo-dtm-trastuzumab with [^{89}Zr]Zr^{4+}at varying concentrations of bioconjugate

The radiolabelling protocol above was repeated at bioconjugate concentrations of 16.7 µM, 3.33 µM, 333 nM, 33 nM, and 3 nM. Radiochemical yields were assessed by ITLC.

Serum stability and target receptor affinity of [^{64}Cu]Cu-sar-dtm-trastuzumab and [^{89}Zr]Zr-dfo-dtm-trastuzumab

Serum stability of [^{64}Cu]Cu-sar-dtm-trastuzumab

A sample of [^{64}Cu]Cu^{2+} (6 MBq in 60 µL of 0.1 M ammonium acetate) was added to sar-dtm-trastuzumab (20 µL, ~230 µg, 80 µM, in 0.1 M NH$_4$OAc), mixed and left at room temperature for 5 min. Radiochemical yield was assessed using ITLC and size exclusion HPLC. A sample of this radiolabelled solution was added to serum, in a

ratio of one part radiolabelled solution to four parts serum (by volume), and incubated at 37 °C. Aliquots were taken for analysis by size exclusion HPLC at 0 h, 20 h, and 40 h.

Serum stability of [^{89}Zr]Zr-dfo-dtm-trastuzumab

A sample of [^{89}Zr]Zr^{4+} (5 MBq, 25 µL, in 0.2 M HEPES) was added to dfo-dtm-trastuzumab (160 µL, 81 µM, in 0.1 M ammonium acetate), mixed and left at room temperature for 10 min. Radiochemical yield was assessed using ITLC and size exclusion HPLC. A sample of this radiolabelled solution was added to serum at incubated at 37 °C. For serum stability experiments at 5 min, 2 days, and 4 days one part radiolabelled solution was added to four parts serum (by volume). For the longest time-point, one part radiolabelled solution was added to two parts serum (by volume) to ensure a sufficient amount of radioactivity was present within the sample for analysis after 7 days incubation. Samples were analysed by size exclusion HPLC at 5 min, 2 days, 4 days and 7 days.

SKOV3 in vitro binding study of [^{64}Cu]Cu-sar-dtm-trastuzumab

SKOV3 cells were grown in T150 flasks in culture medium. Cells were aspirated and washed with PBS, then treated with trypsin to detach the cells from the flask and then washed again with PBS. The cells were resuspended in HBSS with 0.2 % BSA to give 2 × 10^5 cells per tube in 250 µL. Trastuzumab solution (500 µL, 2 µM – 1 nM, in HBSS with 0.2 % BSA) was added to each tube, followed by [^{64}Cu]Cu-sar-dtm-trastuzmab (250 µL, 1 µg, 10 kBq, in HBSS with 0.2 % BSA). Tubes were incubated on ice for 1 hr. The cells were washed by centrifuging at 500 rcf for 3 min, aspirating the supernatant, and resuspending in ice cold PBS (1 mL), centrifuging once more at 500 rcf for 3 min, and aspirating the supernatant. The radioactivity associated with cell pellets was counted using a Wallac 1282 Compugamma Universal Gamma Counter. Non-linear regression analysis was performed using a one site total binding fit model (Graphpad Prism 8).

In vivo assessment of [^{64}Cu]Cu-sar-dtm-trastuzumab

Biodistribution study of [^{64}Cu]Cu-sar-dtm-trastuzumab

The biodistribution of [^{64}Cu]Cu-sar-dtm-trastuzumab was assessed in twelve 9-week-old NOD scid gamma (NSG) mice, which were purchased from Charles River UK and left to acclimatise for five days before use with *ad libitum* access to water and diet (PicoLab Rodent Diet 20, LabDiet, St Louis, Missouri). Mice were housed in cages of 6 equipped with Lignocel soft wood pellets, environmental enrichment, and maintained at a temperature of 20 °C to 24 °C and 45% to 65 % humidity. For injection, mice were anaesthetised by inhalation of a 3% isoflurane-oxygen mixture and placed on a nose cone for maintenance with a 1.5-2.5 % isoflurane-oxygen mixture. The mice were injected intravenously (tail vein) with ~1.5 MBq (150 µg, 150 µL in 0.9 % saline). Mice were housed individually in grid floor cages after injection. Biodistribution mice were placed in three groups (n=4) which were culled under anaesthesia at 2 h, 24 h, or 48 hours post injection by cervical dislocation, followed by tail nick to ensure cessation of circulation. Organs were harvested, washed in PBS, dried briefly, and then placed in gamma counter tubes for weighing and radioactivity counting. The radioactivity associated with each tube was counted using a Wallac 1282 Compugamma Universal Gamma Counter. This data was processed to calculate the % injected dose g^{-1} (% ID g^{-1}).

PET imaging study of [^{64}Cu]Cu-sar-dtm-trastuzumab

An *in vivo* PET imaging study was carried out on two 9-week-old NSG mice which were purchased from Charles River U2 K and left to acclimatise for five days before use with *ad libitum* access to water and diet. PET images were acquired in a nanoScan® *in vivo* pre-clinical PET/CT imager (Mediso, Hungary). The mice were anaesthetised by inhalation of a 3% isoflurane-oxygen mixture and placed on a nose cone for maintenance with a 1.5-2.5 % isoflurane-oxygen mixture. These mice were then injected intravenously (*via* tail vein) with [^{64}Cu]Cu-

sar-dtm-trastuzumab ~2.5 MBq (250 µg, 140 µL in 0.9 % saline). PET acquisition was started at 1h post-injection (60 min scan) and scans were repeated at 23 h (60 min scan), and 46 h (120 min scan) post injection. The scanner bed was maintained at 37 °C, and respiration monitoring was conducted throughout the scan. Scans were acquired at 1:5 coincidence mode and 5-ns coincidence time window. Images were reconstructed using Tera-Tomo 3D reconstruction (Mediso) with 4 iterations, 6 subsets, 1–3 coincidence mode, voxel sized 0.4 mm (isotropic), energy window 400–600 keV with attenuation and decay correction.

Ex vivo serum stability

Upon confirmation of death, ~200 µL of blood was harvested from each mouse by cardiac puncture. One drop was placed aside for biodistribution study whilst the rest was placed in a vet tube with gentle agitation and set aside for 1 hour. Vet tubes were centrifugated at 4000 rcf for 12 minutes. The serum was aspirated and analysed by size exclusion HPLC and SDS-PAGE (as described above).

Figures

(a)

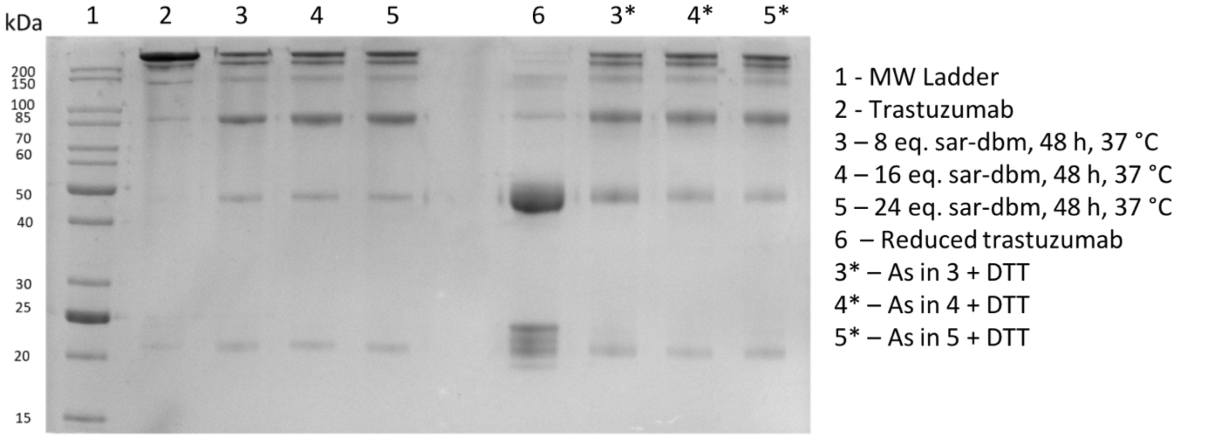

(b)

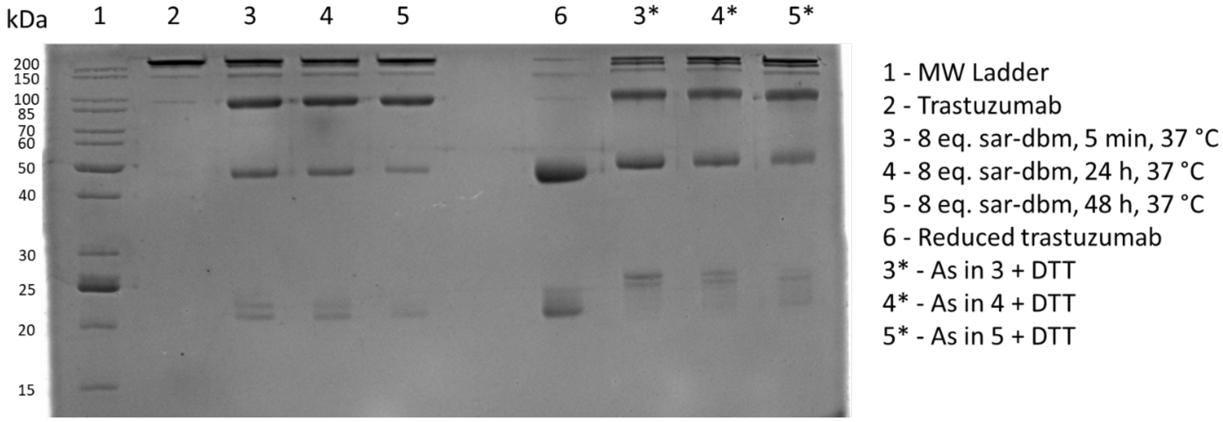

Figure S1. (a) The conjugation reaction between reduced trastuzumab and varying equivalents of sar-dbm was monitored by SDS-PAGE. Conjugation products were also analysed using *reducing* SDS-PAGE conditions: dithiothreitol (DTT) was incubated with the antibody conjugate prior to running the SDS-PAGE. Using 8 equivalents of sar-dbm resulted in dithiol re-bridging. With increasing equivalents of sar-dbm, improvements were negligible. Comparison of lanes 3-5 with lanes 3*-5* indicated that the bioconjugates were stable towards DTT mediated degradation. **(b)** The conjugation reaction between reduced trastuzumab and sar-dbm was monitored over time by SDS-PAGE. Thiol substitution to give bridged dithiomaleimide conjugates proceeded rapidly (within 5 min, lane 3), as evidenced by the appearance of re-bridged "full" and "half" antibody constructs. Increased incubation time (24 – 48 h, lanes 4, 5) did not significantly increase the amounts of these species. However, full hydrolysis of dithiomaleimide conjugates to stable dithiomaleamic acid conjugates required > 5 min (Lane 3*); to ensure full hydrolysis, the conjugate was incubated for 48 h at 37 °C and pH 8.5 (lanes 4*, 5*).

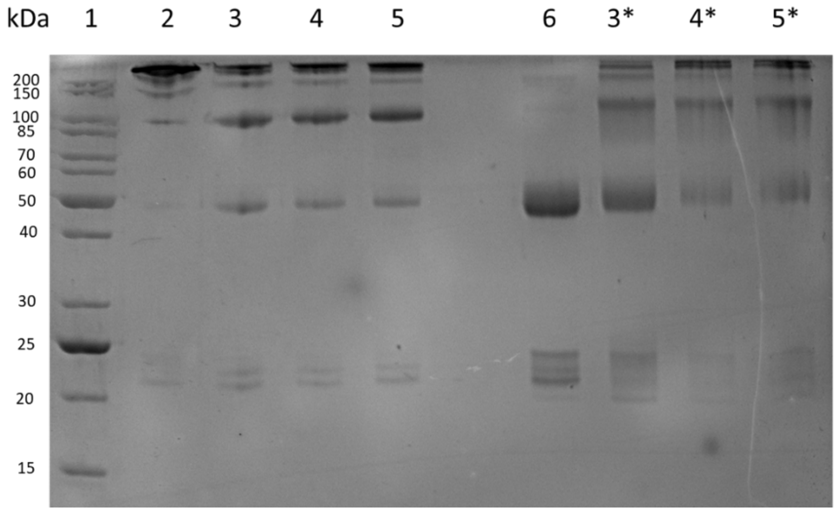

Figure S2. The conjugation reaction between reduced trastuzumab and dfo-dbm was monitored over time by SDS-PAGE (lanes 3-5). Conjugation products were also analysed using *reducing* SDS-PAGE conditions: dithiothreitol (DTT) was incubated with the antibody conjugate prior to running the SDS-PAGE (lanes 3*-5*). Thiol substitution to give bridged dithiomaleimide conjugates proceeded rapidly (within 5 min, lane 3), as evidenced by the appearance of re-bridged “full” and “half” antibody constructs. Increased incubation time (1 h – 2 h, lanes 4, 5) did not significantly increase the amounts of these species. However, full hydrolysis of dithiomaleimide conjugates to stable dithiomaleamic acid conjugates required > 5 min (lane 3*); to ensure full hydrolysis, the conjugate was incubated for 2 h at 37 °C and pH 8.5 (lanes 4*, 5*).

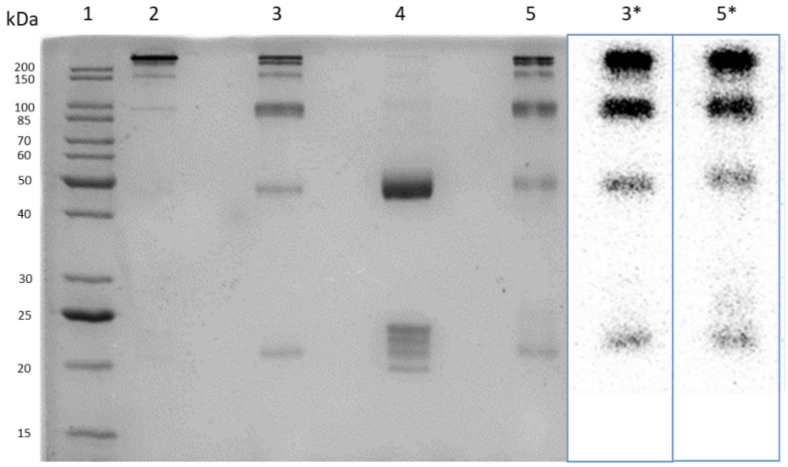

Figure S3. SDS-PAGE bright light image (left) and autoradiography image (right) of [^{64}Cu]Cu-sar-dtm-trastuzumab. The radioactivity signal from the radiolabelled immunoconjugate was coincident with the stained protein bands corresponding to “full” and “half” antibody (lanes 3, 3*). The radiolabelled bioconjugate was stable under reducing conditions where 100 equivalents of DTT were added, as shown by the coincident radioactivity signal and stained protein bands (lanes 5 and 5*).

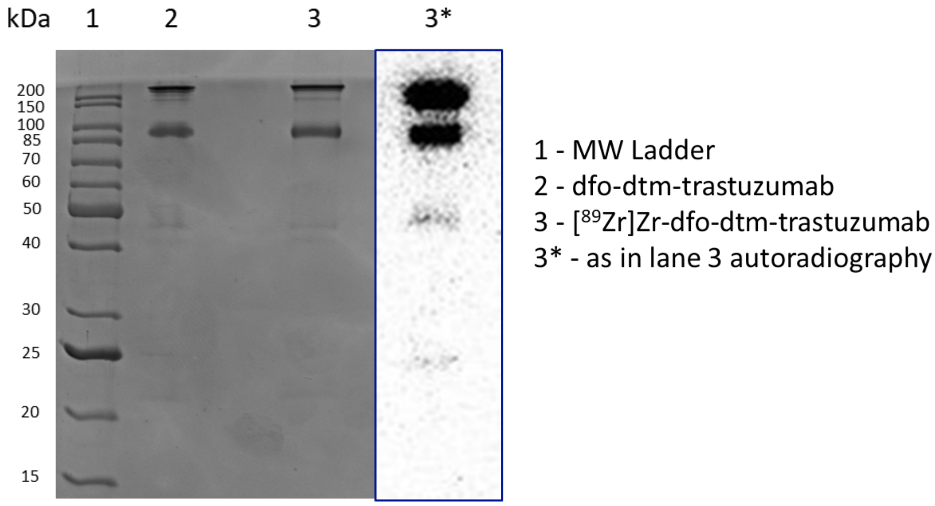

Figure S4. SDS-PAGE bright light image (left) and autoradiography image (right) of [^{89}Zr]Zr-dfo-dtm-trastuzumab. The radioactivity signal from the radiolabelled immunoconjugate was coincident with the stained protein bands corresponding to “full” and “half” antibody.

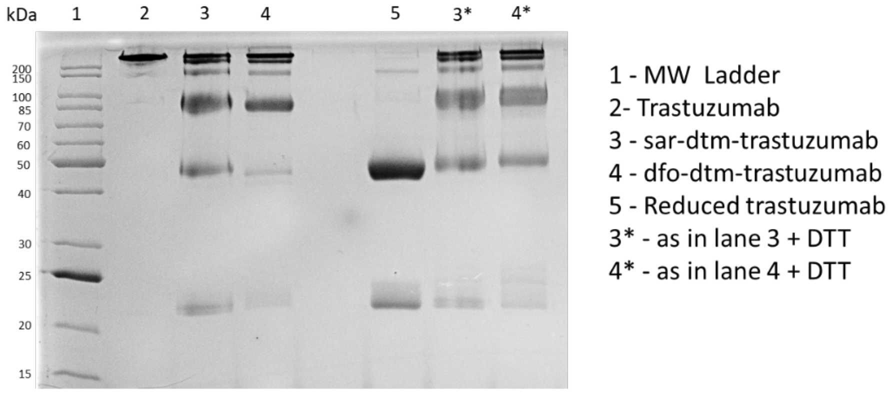

Figure S5. SDS-PAGE of sar-dtm-trastuzumab and dfo-dtm-trastuzumab (lanes 3 and 4). Dithiomaleimide hydrolysis was complete within 48 h at 37 °C as shown by the bioconjugates’ stability under denaturing conditions in the presence of 100 equivalents of DTT (lanes 3* and 4*).

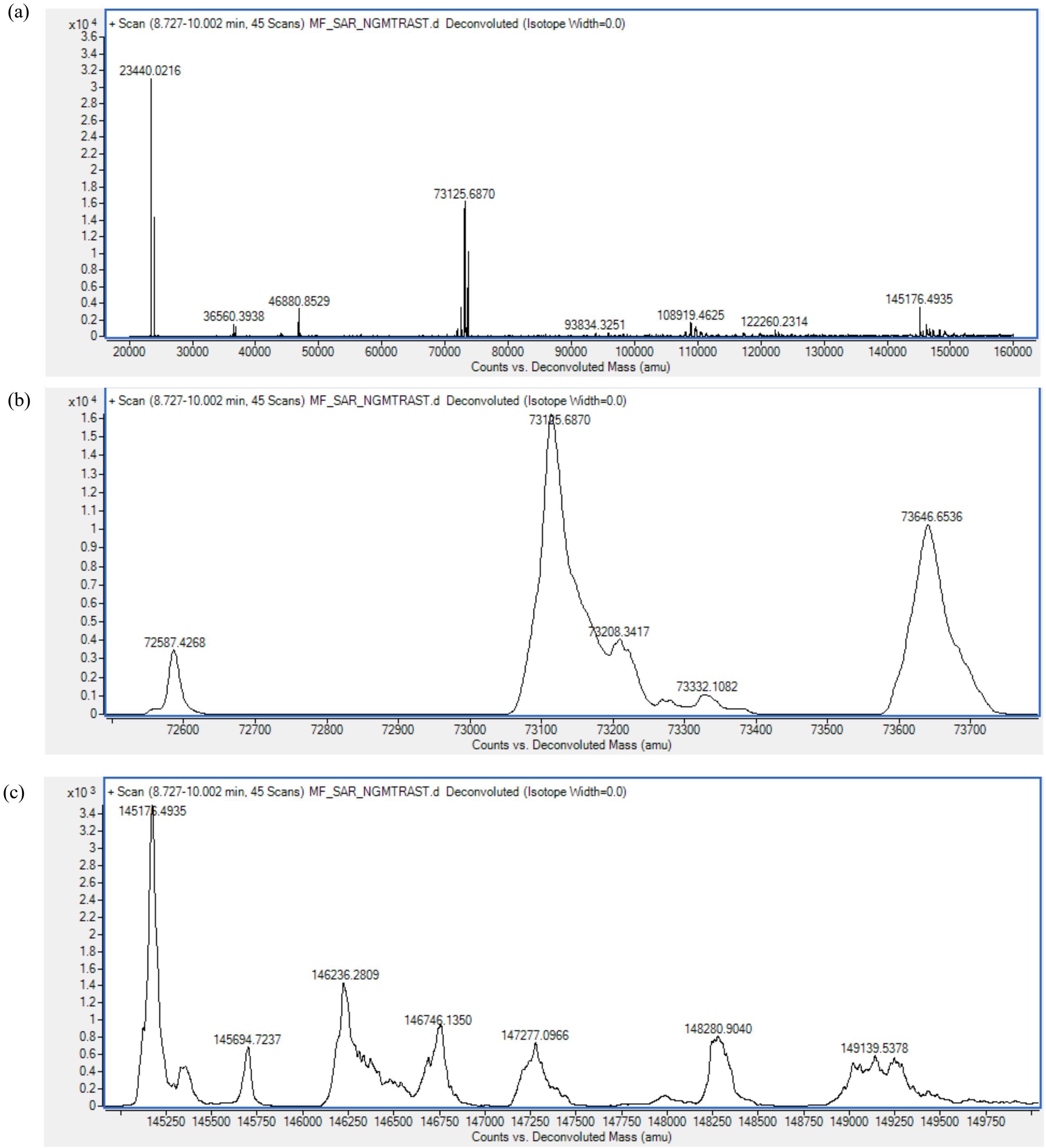

Figure S6. High resolution ESI deconvoluted mass spectrum of sar-dtm-trastuzumab: (a) full spectrum, (b) expanded view of the region associated with "half" antibody conjugates, and (c) expanded view of the region associated with "full" antibody conjugates (see Table S2 for assignments, page 6).

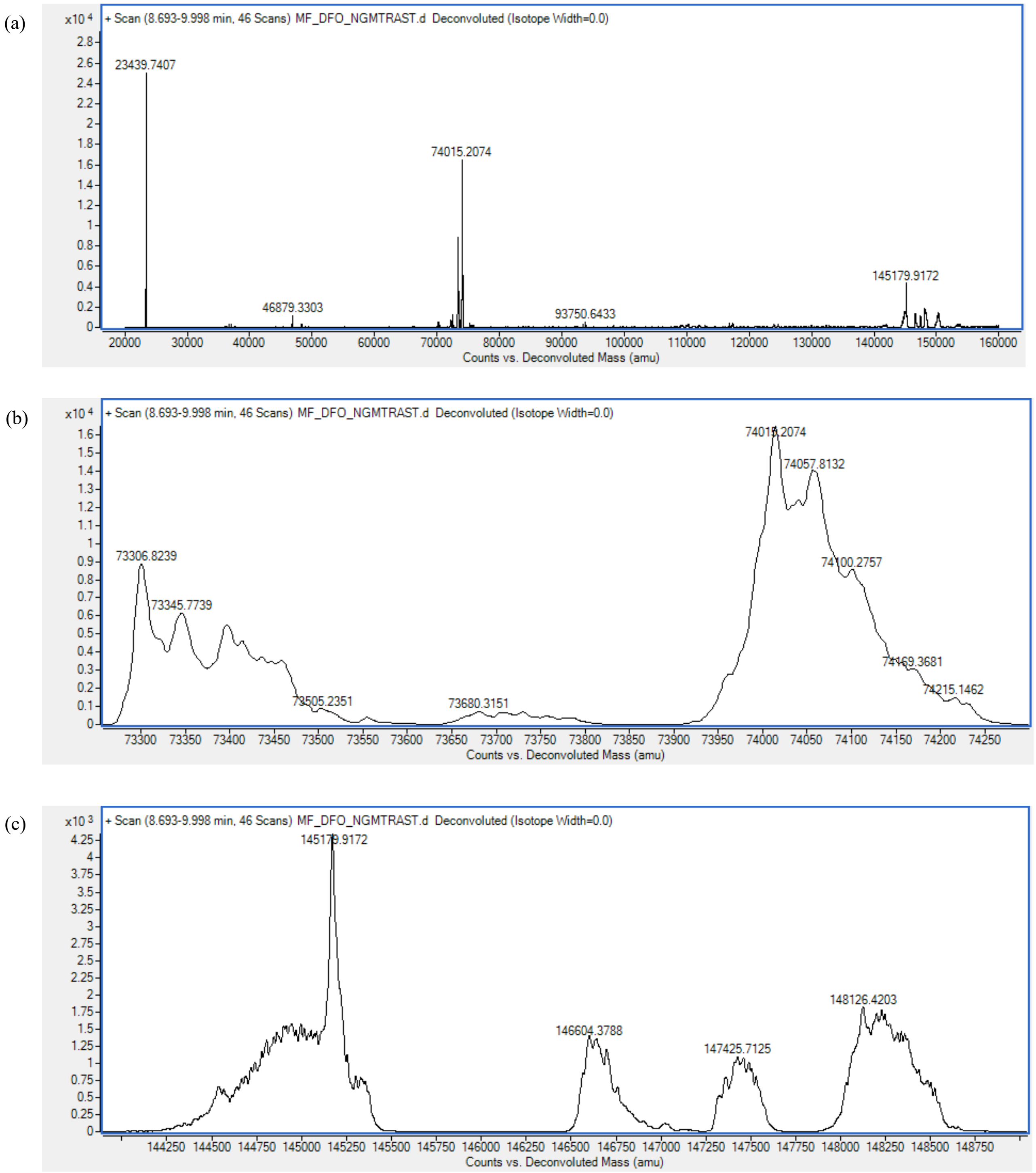

Figure S7. High resolution ESI deconvoluted mass spectrum of dfo-dtm-trastuzumab: (a) full spectrum, (b) expanded view of the region associated with “half” antibody conjugates, and (c) expanded view of the region associated with “full” antibody conjugates (see Table S2 for assignments, page 6).

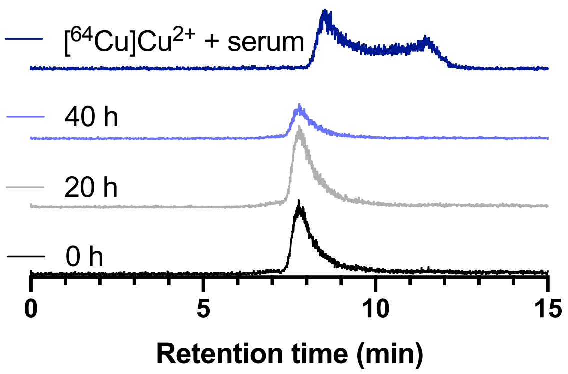

Figure S8. Size exclusion HPLC chromatograms of [^{64}Cu]Cu-sar-dtm-trastuzumab after incubation with human serum for 20 h and 40 h. Each chromatogram shows a single radioactive signal, with the retention time of each signal corresponding to [^{64}Cu]Cu-sar-dtm-trastuzumab (retention time = 7.8 min). When [^{64}Cu]Cu^{2+} was incubated with human serum for 20 h at 37 °C, two signals were observed: the first signal at 8.5 min corresponds to [^{64}Cu]Cu^{2+} complexed by serum protein; the second peak at 11.6 min corresponds to a lower molecular weight species or unreacted [^{64}Cu]Cu^{2+}.

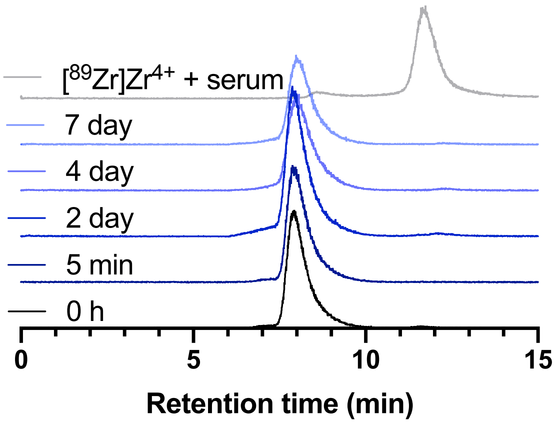

Figure S9. Size exclusion HPLC chromatograms of [^{89}Zr]Zr-dfo-dtm-trastuzumab after incubation with human serum, over the course of 7 days. Each chromatogram shows a single radioactive signal, with the retention time of each signal corresponding to [^{89}Zr]Zr-dfo-dtm-trastuzumab (retention time = 7.8 min). When [^{89}Zr]Zr^{4+} was incubated with human serum, two signals were observed: the first signal at 8.5 min corresponds to [^{89}Zr]Zr^{4+} complexed by serum protein; the second peak at 11.8 min corresponds to a lower molecular weight species or unreacted [^{89}Zr]Zr^{4+}.

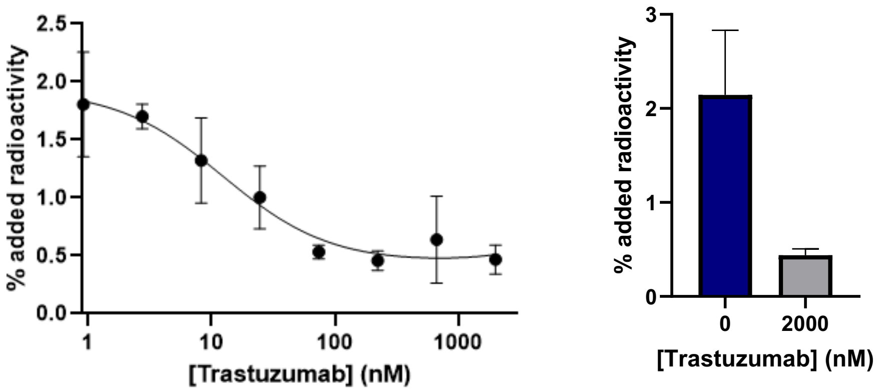

Figure S10. Cell binding study of [^{64}Cu]Cu-sar-dtm-trastuzumab with HER2-positive SKOV3 cells. [^{64}Cu]Cu-sar-dtm-trastuzumab binds to HER2-positive SKOV3 cells, and this can be inhibited by increasing concentrations of native trastuzumab. Here, the % of added [^{64}Cu]Cu-sar-dtm-trastuzumab radioactivity associated with the cell pellet is plotted against the concentration of added trastuzumab. A bar chart has been used to show the difference in cell uptake of [^{64}Cu]Cu-sar-dtm-trastuzumab at the lowest (0 nM) and highest (2000 nM) trastuzumab concentrations tested. Error bars show standard deviation. Binding studies were carried out in experimental triplicate (n=3, n=3, n=2).

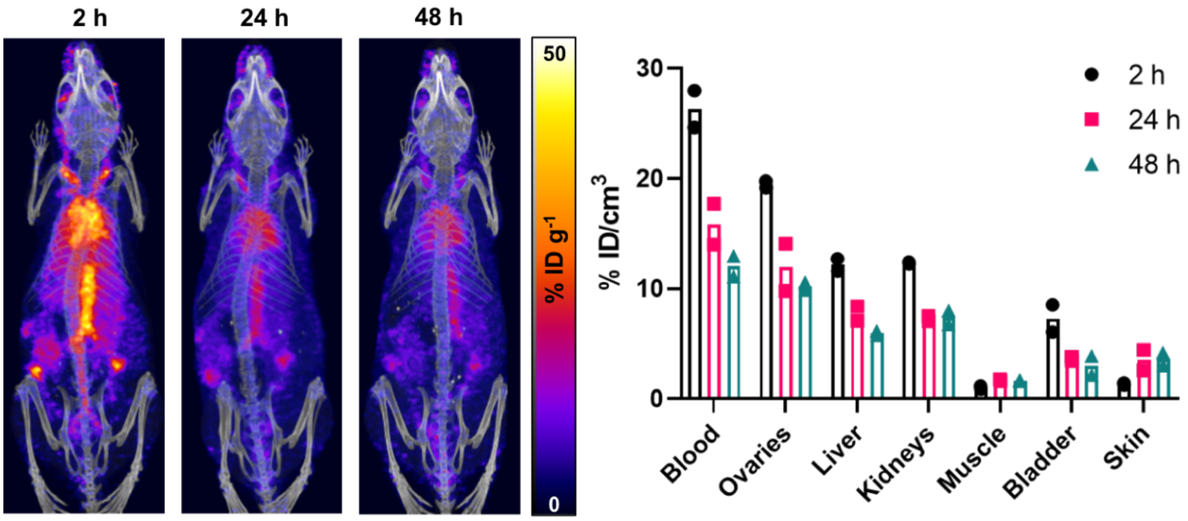

Figure S11. Left, maximum intensity projections of mice injected with [^{64}Cu]Cu-sar-dtm-trastuzumab at 2 h, 24 h, and 48 h post injection. Right, regions of interest were selected on VivoQuant (inviCRO, LLC, Boston, USA), and percentage injected dose per millilitre (% ID/mL) were calculated for each. Bars show mean values (n=2), individual data points are plotted.

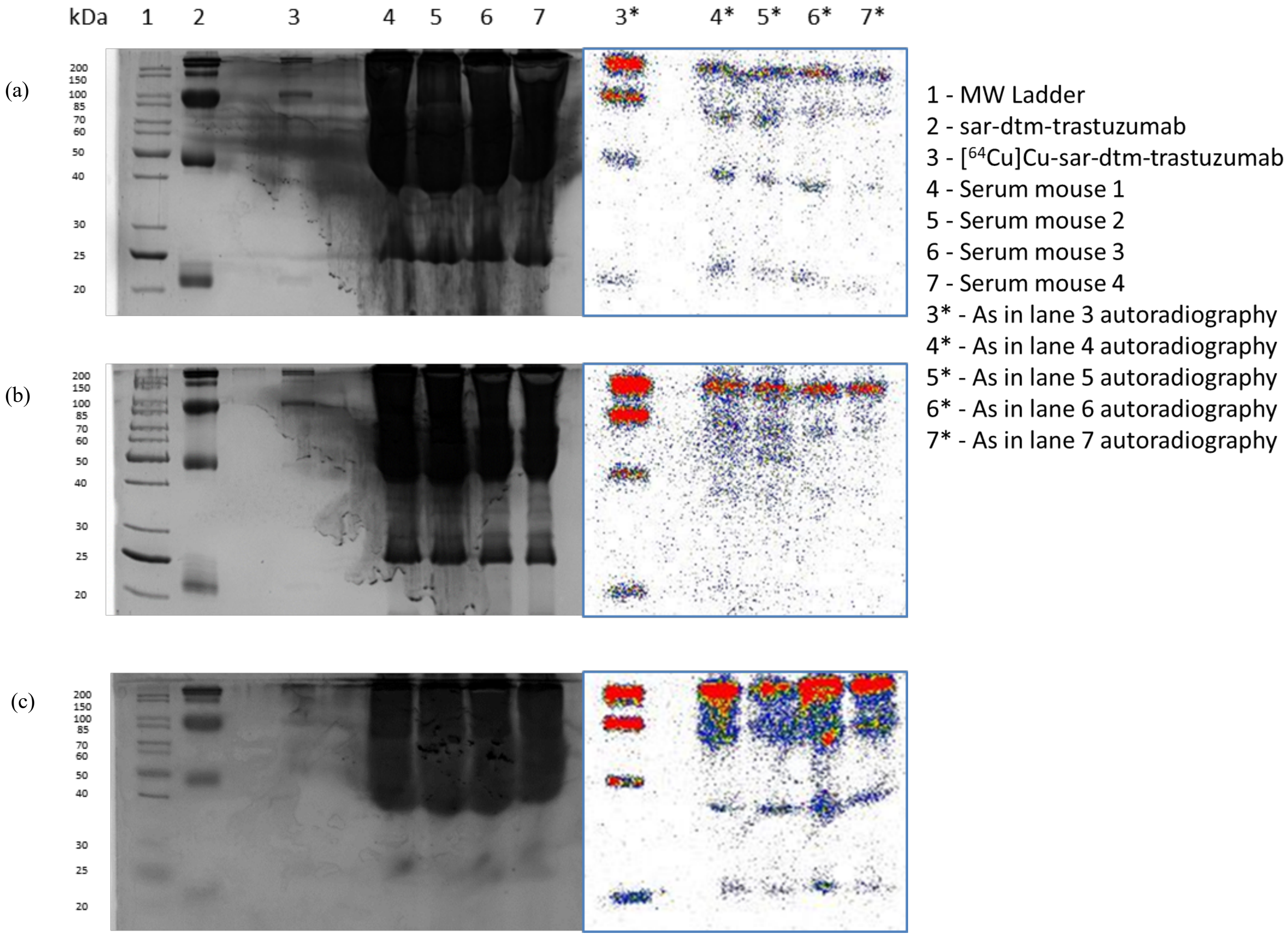

Figure S12. SDS-PAGE analysis of mouse serum after injection with [^{64}Cu]Cu-sar-dtm-trastuzumab, (left) bright light images, (right) autoradiography images. (a) 2 h post-injection, (b) 24 h post-injection, (c) 48 h post-injection.

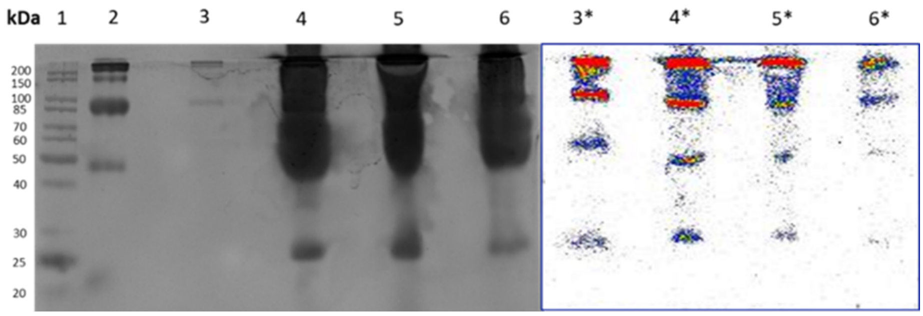

1 - MW Ladder
2 - sar-dtm-trastuzumab
3 - [^{64}Cu]Cu-sar-dtm-trastuzumab
4 - Mouse serum 2 h post-injection
5 - Mouse serum 24 h post-injection
6 - Mouse serum 48 h post-injection
3* - As in lane 3 autoradiography
4* - As in lane 4 autoradiography
5* - As in lane 5 autoradiography
6* - As in lane 6 autoradiography

Figure S13. SDS-PAGE of ex-vivo serum samples, bright light image (left) and autoradiography image (right).

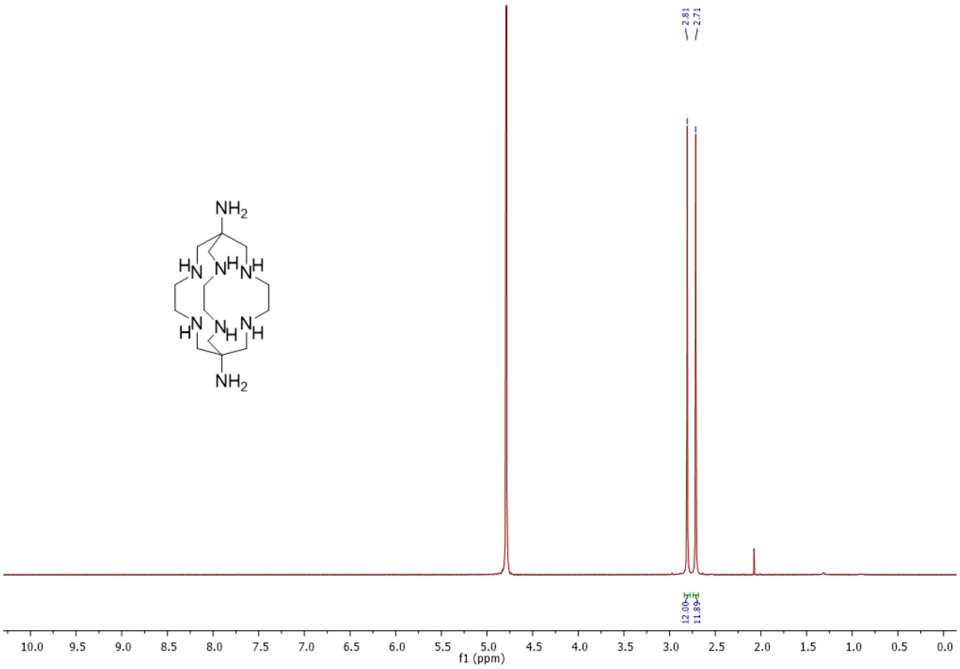

Figure S14. ^{1}H NMR spectrum for $(NH_2)_2$sar (D_2O, 400 MHz).

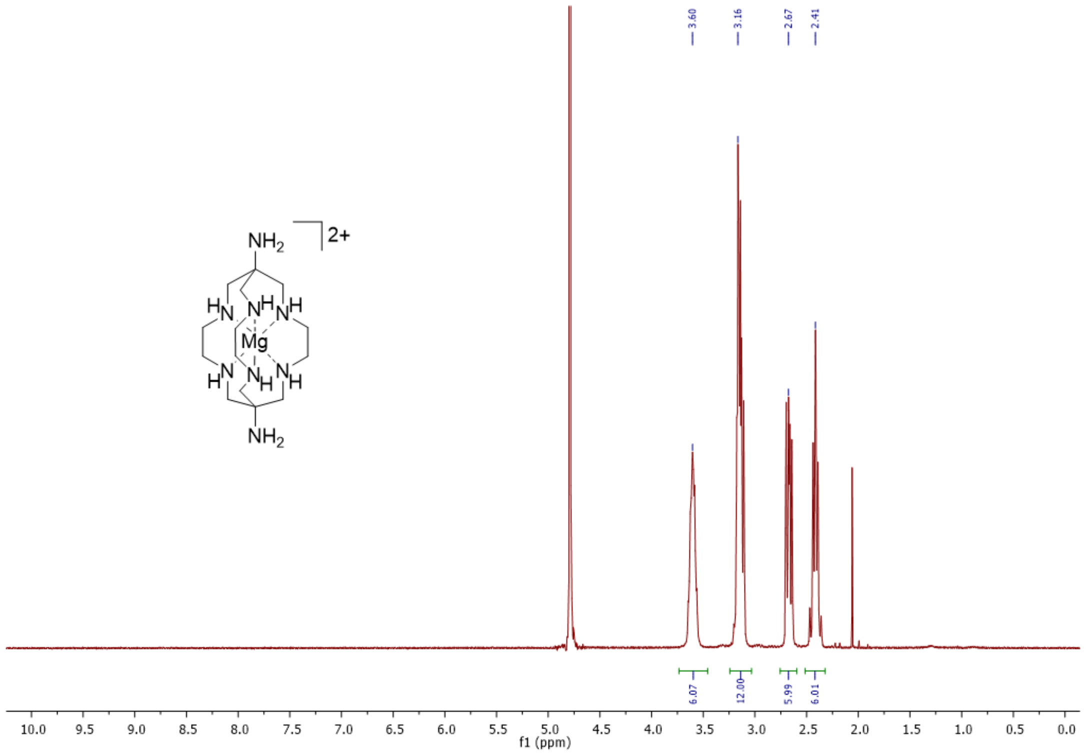

Figure S15. ^1H NMR spectrum for $[Mg(NH_2)_2sar](CF_3SO_3)_2$ (D_2O, 400 MHz).

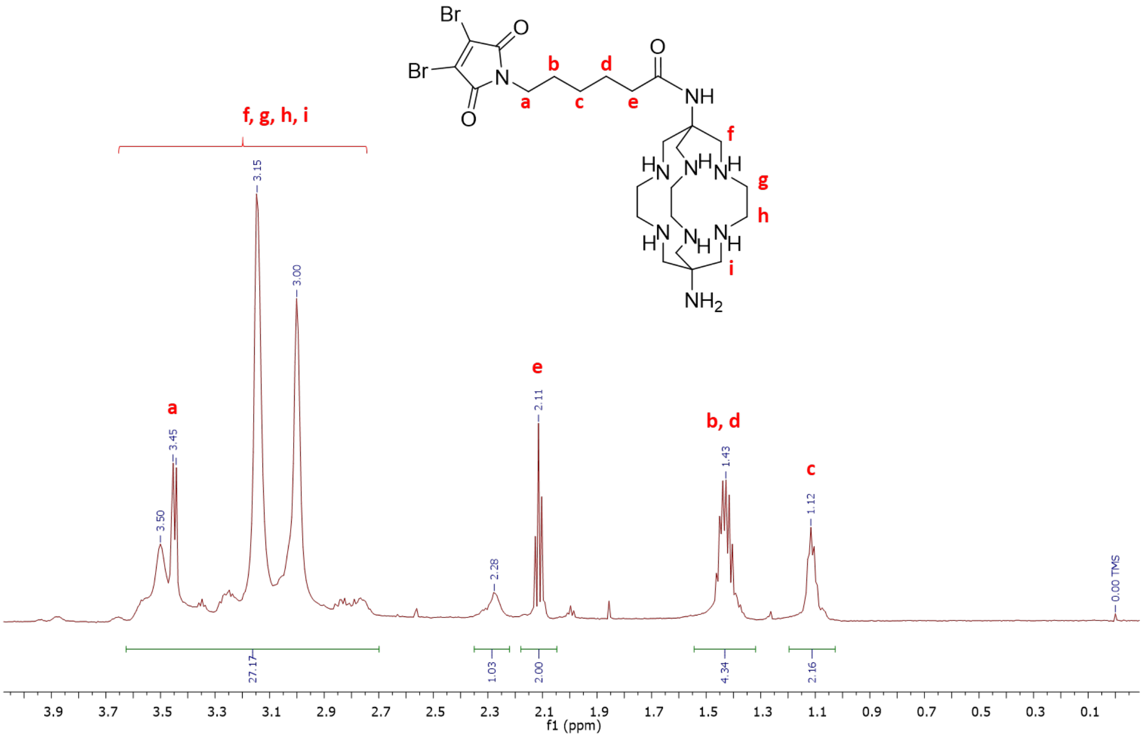

Figure S16. ^1H NMR spectrum for sar-dbm (**3**) (D_2O, 700 MHz).

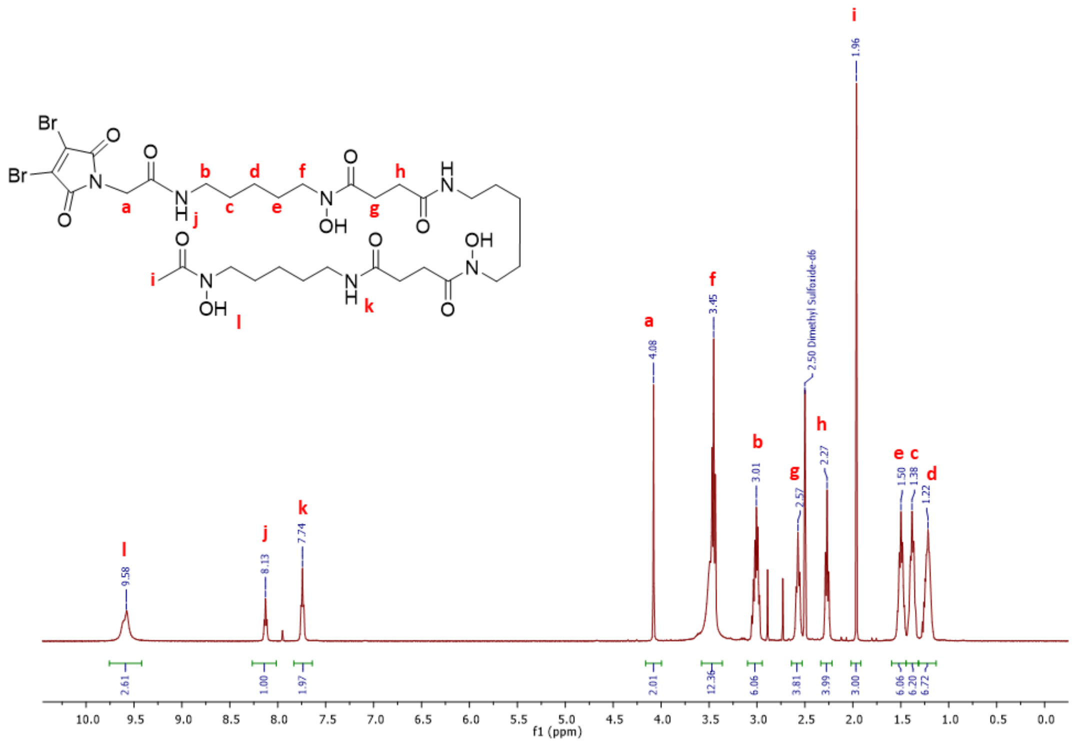

Figure S17. ^{1}H NMR spectrum for dfo-dbm (**6**) (DMSO-d_6, 400 MHz). The similar chemical environment of the atoms along the DFO pentyl and ethyl chains resulted in coincident resonances at 400 MHz.

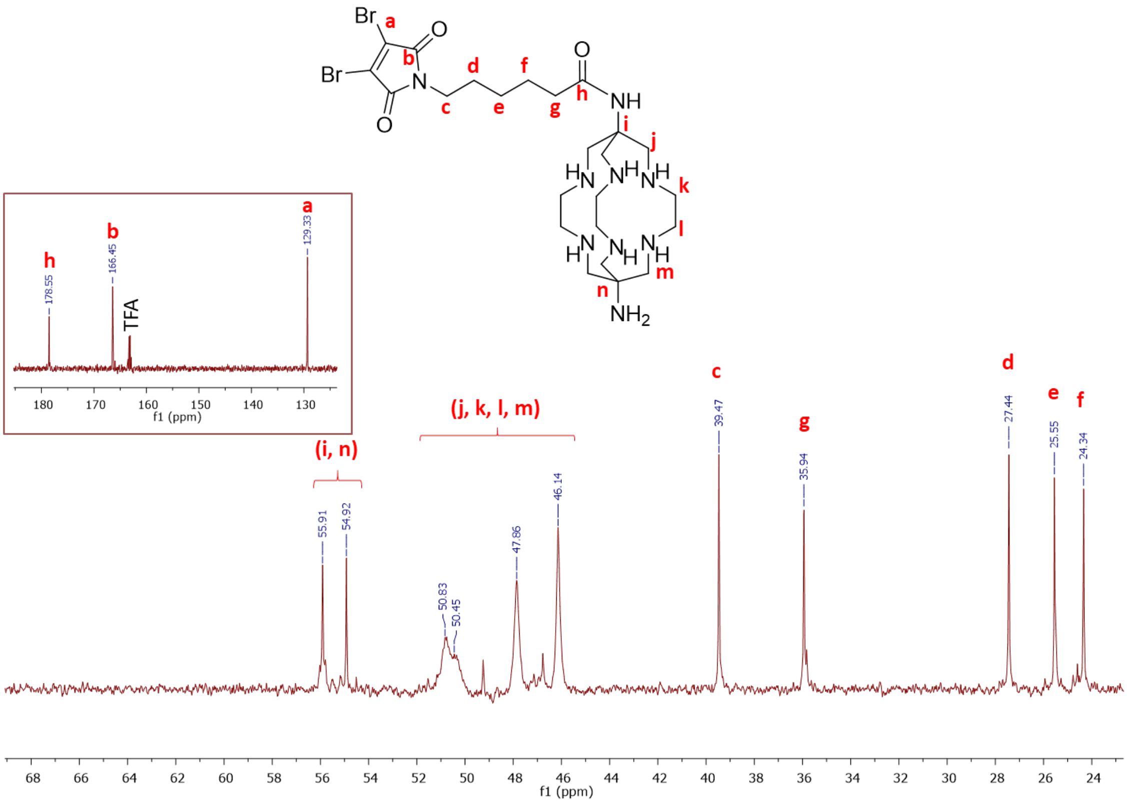

Figure S18. ^{13}C NMR spectrum for sar-dbm (**3**) (D_2O, 175 MHz).

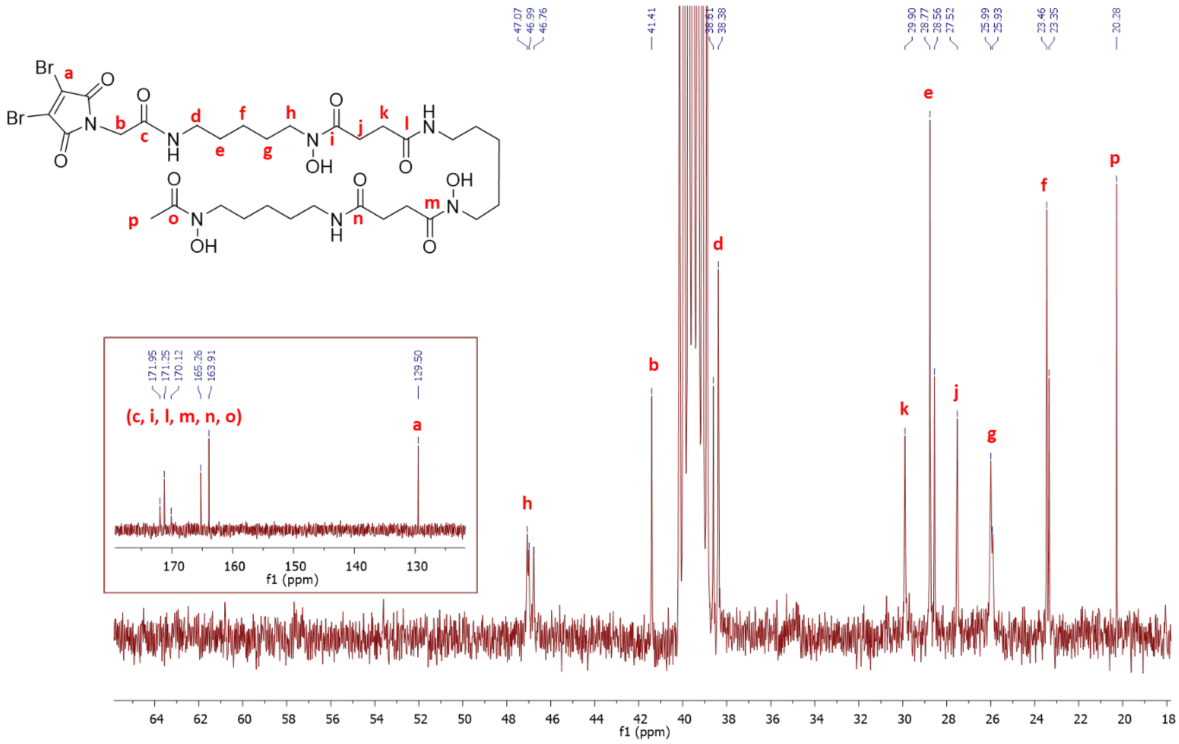

Figure S19. ^{13}C NMR spectrum for dfo-dbm (**6**) (DMSO-d_6, 100 MHz). The similar chemical environment of the atoms along the DFO pentyl and ethyl chains resulted in often coincident, or nearly coincident resonances at 100 MHz.

(a)

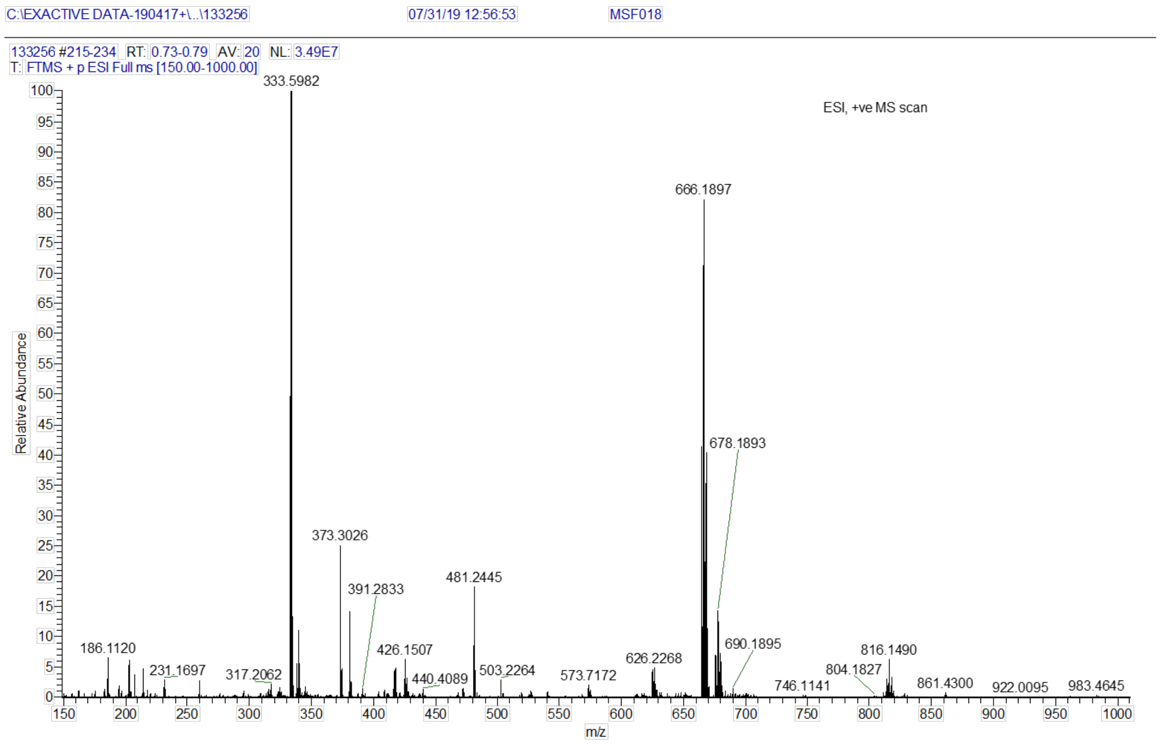

(b)

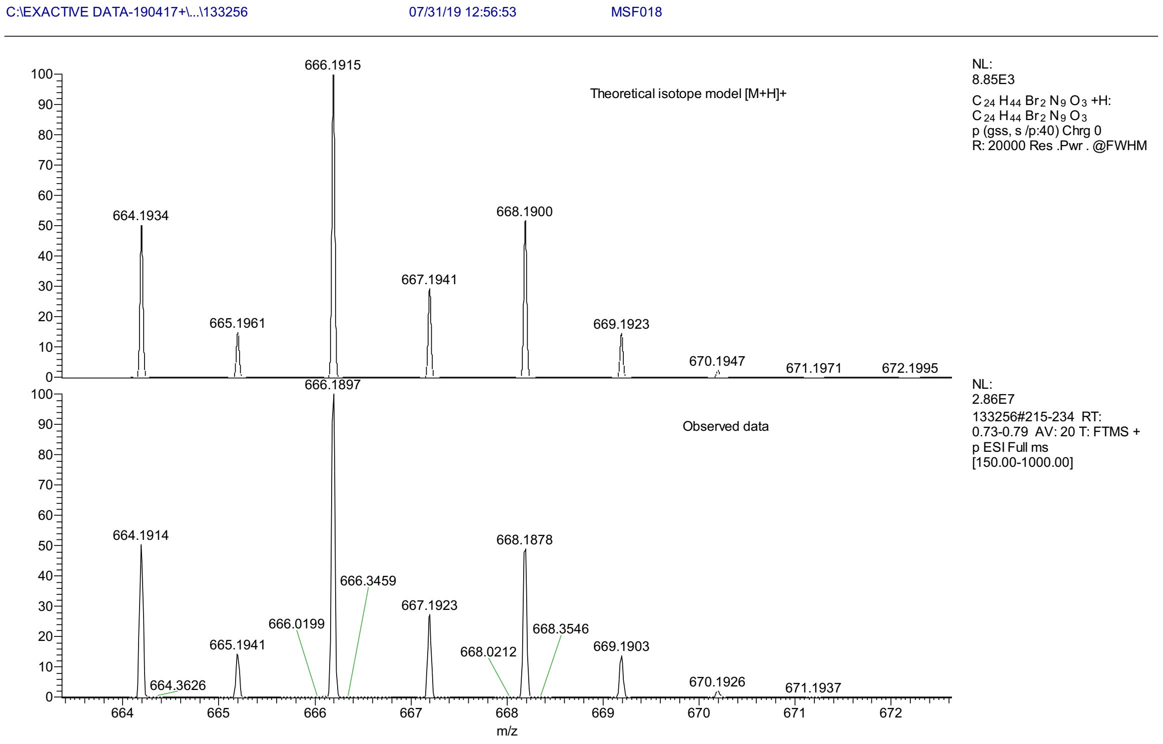

Figure S20. High-resolution ESI-mass spectrum for sar-dbm: (a) full mass spectrum; (b) expanded view of the $[MH]^+$ signal showing (top) calculated isotope pattern, and (bottom) observed data.

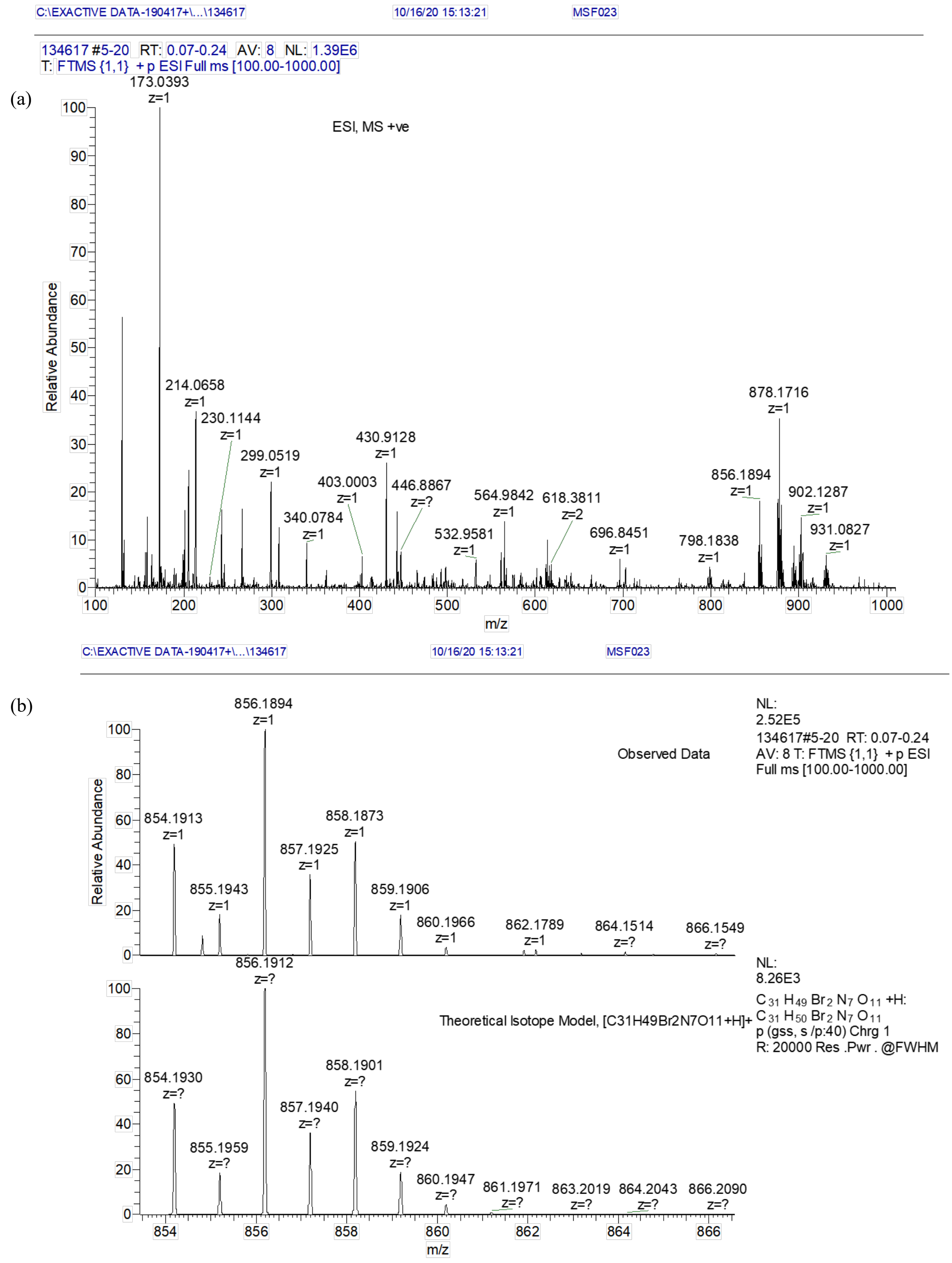

Figure S21. High-resolution ESI-mass spectrum for dfo-dbm: (a) full mass spectrum; (b) expanded view of the $[MH]^+$ signal showing (top) observed data and (bottom) calculated isotope pattern.

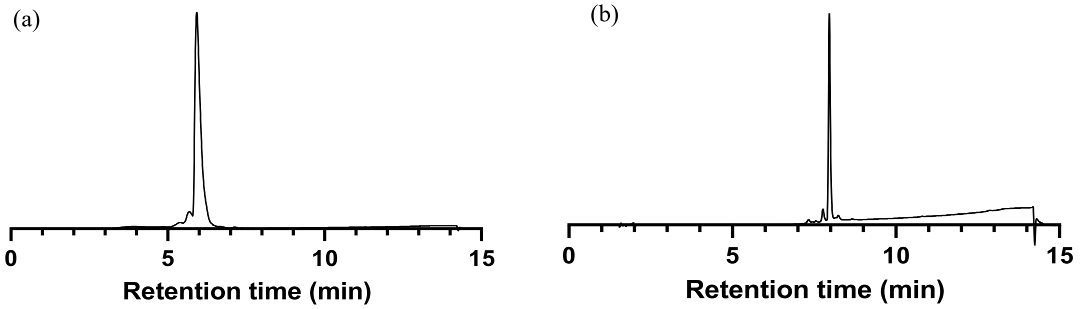

Figure S22. Analytical C18 reverse-phase HPLC chromatograms with UV detection at λ254 of (a) sar-dbm with a retention time of 5.92 min and (b) dfo-dbm with a retention time of 7.95 min. Agilent ZORBAX Eclipse XDB-C18, 80Å, 4.6 x 150 mm, 5 µm. Gradient: 1 mL min^{-1} A = 0.1% FA in water, B = 0.1 % FA in acetonitrile. 0-2 min 5 % B in A, 2-11 min 5 %→95 % B in A, 12-12.10 min 95 %→5 % B in A, 12.10-15 min 5 % B in A.

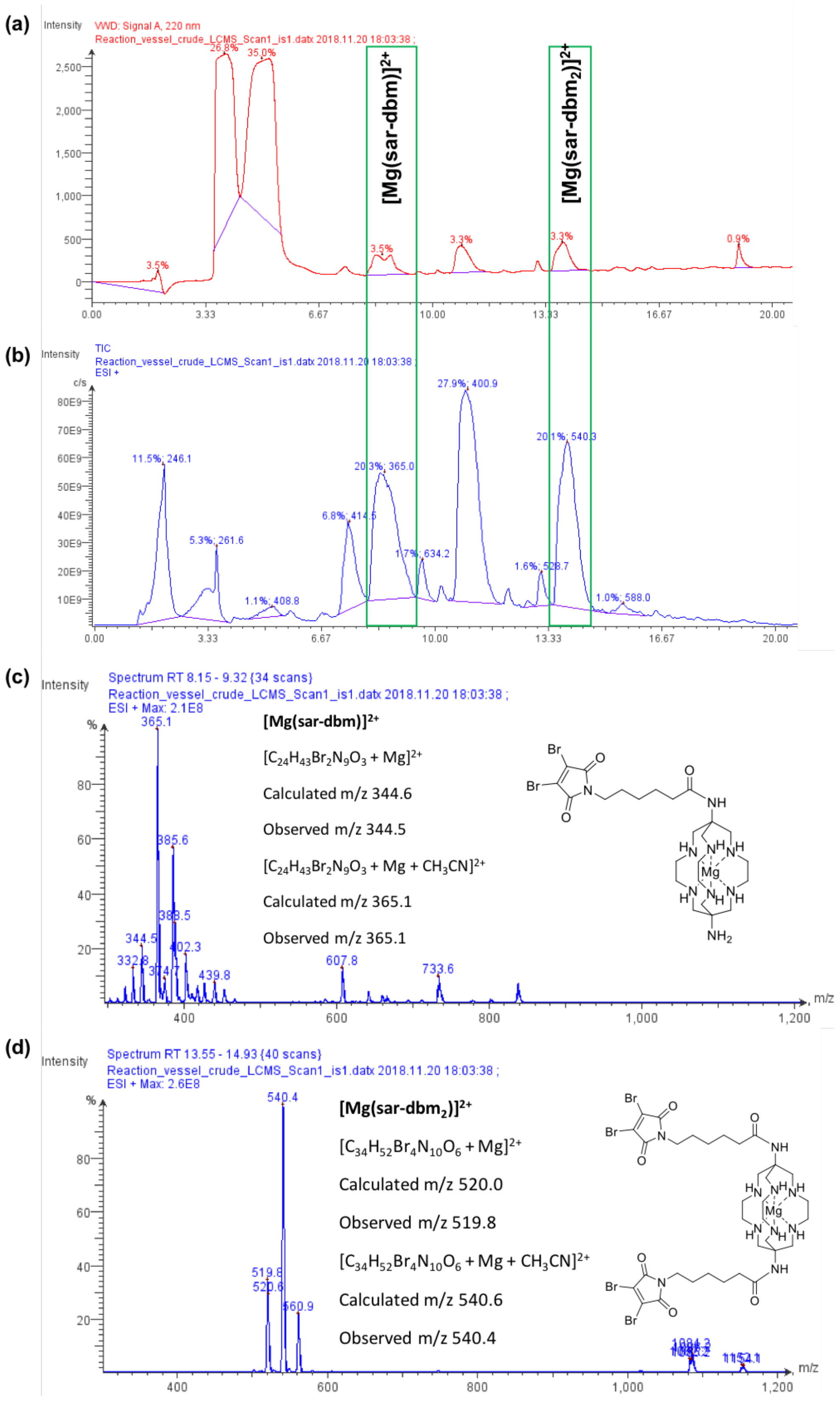

Figure S23. The compound $[Mg(sar\text{-}dbm_2)]^{2+}$ was also observed in reactions containing $[Mg((NH_2)_2sar)]^{2+}$ and 3,4-dibromo-maleimide-*N*-hexanoic acid. In this compound, both apical primary amines of $(NH_2)_2$sar ligand react with two equivalents of 3,4-dibromo-maleimide-*N*-hexanoic acid to yield a sarcophagine chelator containing two dibromomaleimide groups. LC-MS analysis of crude reaction mixtures showed the presence of both sar-dbm and sar-dbm$_2$: (a) UV chromatogram (λ = 220 nm); (b) total ion current chromatogram (positive mode); (c) Mass spectrum of $[Mg(sar\text{-}dbm)]^{2+}$ (retention time = 8.15 min); (d) Mass spectrum of $[Mg(sar\text{-}dbm_2)]^{2+}$ (retention time = 13.55 min).

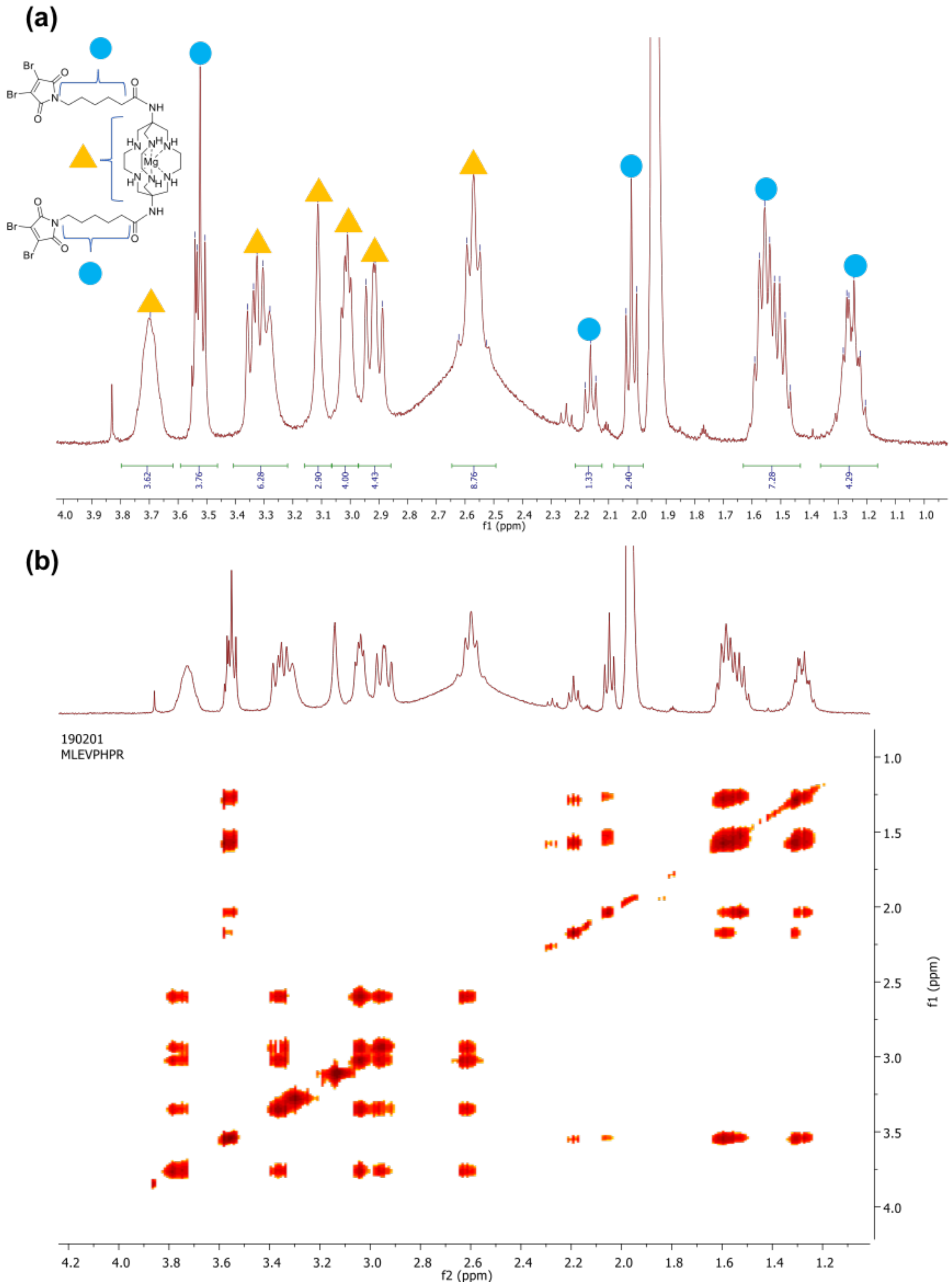

Figure S24. (a) ^{1}H NMR spectrum obtained for isolated $[Mg(sar\text{-}dbm_2)]^{2+}$ (CD_3CN, 400 MHz). (b) ^{1}H TOCSY NMR spectrum obtained for $[Mg(sar\text{-}dbm_2)]^{2+}$ (CD_3CN, 400 MHz). N.B.: This material was isolated from a crude reaction in which Mg^{2+} was *not* removed from sar derivatives, prior to HPLC purification.

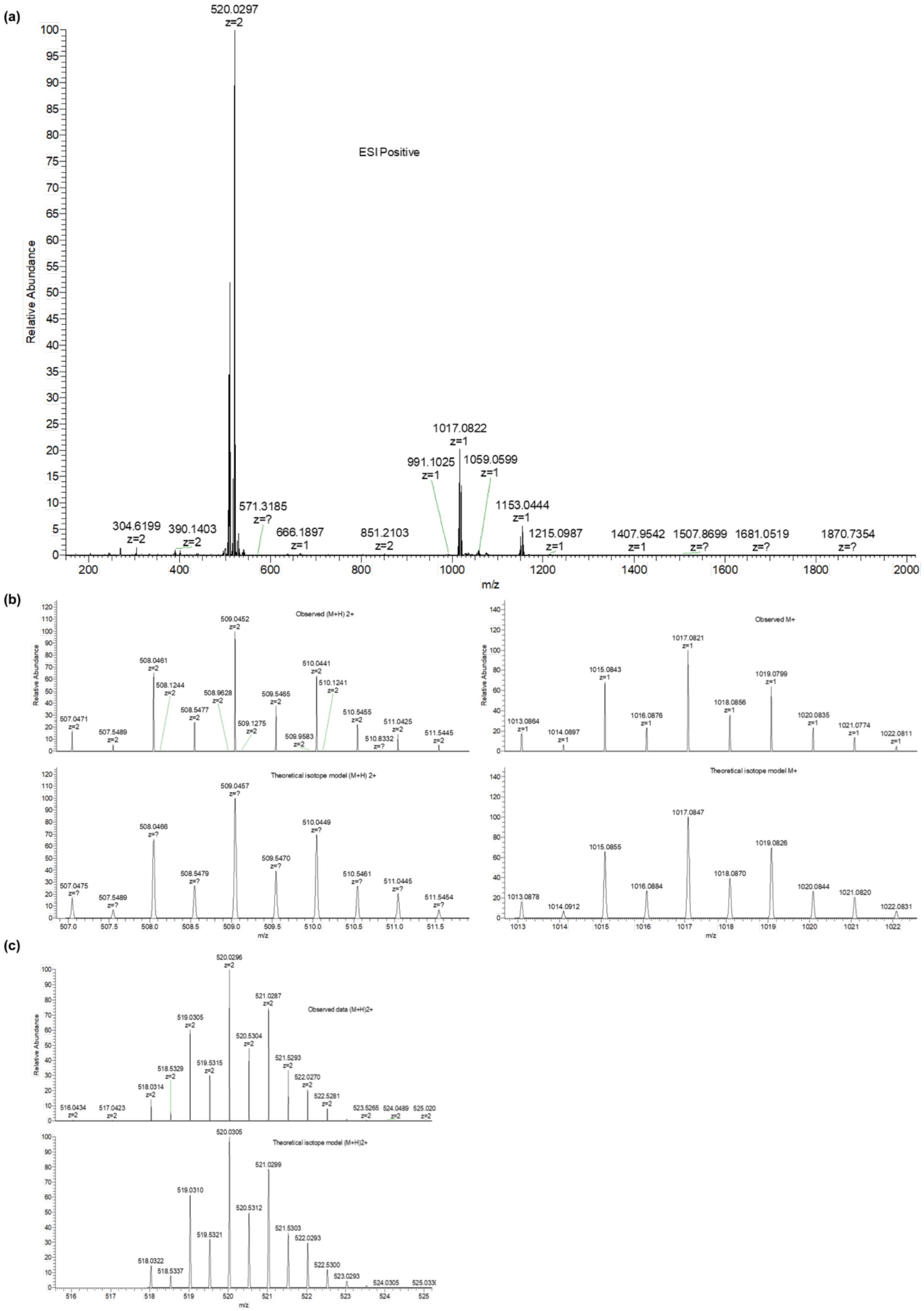

Figure S25. High-resolution ESI-mass spectrum showing signals corresponding to both sar-dbm$_2$ and [Mg(sar-dbm$_2$)]$^{2+}$: (a) full mass spectrum; (b) expanded view of signals corresponding to sar-dbm$_2$ showing (top) observed data and (bottom) theoretical isotope pattern; (c) expanded view of signals corresponding to [Mg(sar-dbm$_2$)]$^{2+}$ showing (top) observed data and (bottom) calculated isotope pattern.

References

1. Geue, R. J., Sargeson, A. M., Hambley, T. W., Snow, M. R., Harrowfield, J. M., (1984) Metal Ion Encapsulation: Cobalt Cages Derived from Polyamines, Formaldehyde, and Nitromethane. *J. Am. Chem. Soc. 106*, 5478–5488.

2. Clark, I. J., Creaser, I. I., Geue, R. J., Hagen, K. S., Lawrance, G. A., Lay, P. A., Sargeson, A. M., Wilne, F. R., (1994) The Synthesis and Structure of Encapsulating Ligands: Properties of Bicyclic Hexamines. *Aust. J. Chem. 47*, 143–179.

3. Paterson, B. M., Buncic, G., McInnes, L. E., Roselt, P., Cullinane, C., Binns, D. S., Jeffery, C. M., Price, R. I., Hicks, R. J., Donnelly, P. S. (2015) Bifunctional ^{64}Cu-Labelled Macrobicyclic Cage Amine Isothiocyanates for Immuno-Positron Emission Tomography. *Dalton Trans.*, *44*, 4901–4909.

4. Morais, M., Nunes, J. P. M., Karu, K., Forte, N., Benni, I., Smith, M. E. B., Caddick, S., Chudasama, V., Baker, J. R. (2017) Optimisation of the Dibromomaleimide (DBM) Platform for Native Antibody Conjugation by Accelerated Post-Conjugation Hydrolysis. *Org. Biomol. Chem. 15*, 2947–2952.

5. Morais, M.; Forte, N.; Chudasama, V.; Baker, J. R. Application of Next-Generation Maleimides (NGMs) to Site-Selective Antibody Conjugation, in *Methods in Molecular Biology;* Massa, S., Devoogdt, N., Eds.; Humana: New York, 2019; Vol. 2033, pp 15–24.

6. Cooper, M. S., Ma, M. T., Sunassee, K., Shaw, K. P., Williams, J. D., Paul, R. L., Donnelly, P. S., and Blower, P. J. (2012) Comparison of ^{64}Cu-Complexing Bifunctional Chelators for Radioimmunoconjugation: Labeling Efficiency, Specific Activity, and *in Vitro* / *in Vivo* Stability. *Bioconjugate Chem. 23*, 1029–1039.

7. Man, F. Khan, A. A., Carrascal-Miniño, A., Blower, P. J., de Rosales, R. T. M. (2020) A Kit Formulation for the Preparation of [^{89}Zr]Zr(oxinate)$_4$ for PET Cell Tracking: White Blood Cell Labelling and Comparison with [^{111}In]In(oxinate)$_3$. *Nucl. Med. Biol. 90–91*, 31–40.
